# mRNA delivery of circumsporozoite protein epitope-based malaria vaccines induces protection in a mouse model

**DOI:** 10.1038/s41541-025-01296-6

**Published:** 2025-11-18

**Authors:** Nelson R. Wu, Nathan Beutler, Xiaozhen Hu, Patrick D. Skog, Alessia Liguori, Yevel Flores-Garcia, Laura Maiorino, Sierra Terada, Danny Lu, Yen-Chung Lai, Justin Ndihokubwayo, Torben Schiffner, Christopher A. Cottrell, Saman Eskandarzadeh, Nushin Alavi, Michael Kubitz, Nicole Phelps, Ryan Tingle, Sam Hodges, John E. Youhanna, Sonya Amirzehni, Darrell J. Irvine, Sunny Himansu, Fidel Zavala, Thomas F. Rogers, Dennis R. Burton, William R. Schief

**Affiliations:** 1 Department of Immunology and Microbiology, The Scripps Research Institute, La Jolla, CA USA; 2 Moderna Inc., Cambridge, MA USA; 3 IAVI Neutralizing Antibody Center, The Scripps Research Institute, La Jolla, CA USA; 4 Johns Hopkins Malaria Research Institute, Department of Molecular Microbiology and Immunology, Bloomberg School of Public Health, Johns Hopkins University, Baltimore, MD USA; 5 Department of Biological Engineering, Massachusetts Institute of Technology, Cambridge, MA USA; 6 Howard Hughes Medical Institute, Chevy Chase, MD USA; 7 Division of Infectious Diseases, Department of Medicine, University of California, San Diego, La Jolla, CA USA; 8 The Ragon Institute of Massachusetts General Hospital, Massachusetts Institute of Technology and Harvard University, Cambridge, MA USA

**Keywords:** Protein vaccines, RNA vaccines

## Abstract

Malaria is a leading cause of disease in developing countries. The licensed malaria vaccines (RTS,S/AS01 and R21/Matrix-M) have shown significant efficacy in human phase 3 trials. Vaccination with radiation-attenuated sporozoites has achieved high levels of protection against malaria in controlled infection studies, although protection was more moderate in clinical trials conducted in malaria-endemic areas. RTS,S/AS01, and R21/Matrix-M contain the repeating NANP motif and the C-terminal domain of the dominant surface circumsporozoite protein (CSP) of *Plasmodium falciparum* (Pf) sporozoites, but do not include the CSP N-terminal domain or epitopes in the junctional region between the N-terminal domain and the NANP repeats. In pursuit of a second-generation malaria *PfCSP* vaccine that surpasses the protection elicited by attenuated sporozoites and current subunit vaccines, we developed self-assembling nanoparticle immunogens each displaying one or more of four different classes of *PfCSP* epitope regions: NANP-repeat epitopes, junctional region-repeat epitopes, and epitopes from the N-terminal and C-terminal domains. In a mouse model of malaria infection, immunization with protein nanoparticles displaying different CSP epitope regions showed a reduction in liver burden ranging from minimal to 90%, with N- and C-terminal domains providing little reduction, but a combination of junctional and NANP repeat epitopes providing a strong reduction. mRNA-delivered nanoparticle and membrane-anchored immunogens displaying both the junctional and NANP repeat epitopes were most effective, exhibiting 99% reduction in liver burden and sterilizing immunity from parasitemia in some mice. The mRNA immunogens represent promising candidates for rapid translation to human challenge studies and could be combined with T cell vaccines to comprise a potential next-generation malaria vaccine.

## Introduction

Malaria is a significant global public health challenge with approximately 249 million cases and an estimated 608,000 deaths in 2022^1^. The tropical disease is caused by *Plasmodium* parasites of which *falciparum* is the cause of most malaria-related mortality^2^. Following transmission of parasites to humans after a bite of a female *Anopheles* mosquito, symptoms such as fever, chills, diarrhea, and convulsions develop within 10 days to 4 weeks^3^. Insecticide-treated nets and anti-malarial drugs have contributed to a decline in malaria cases, but increasing drug resistance by malaria parasites has stymied eradication efforts, highlighting the need for effective malaria vaccines^4^.

*Plasmodium* parasites undergo a complex life cycle between the human host and mosquito vector. There has been considerable vaccine development focused on disrupting the parasite life cycle at various stages, specifically aimed at preventing hepatocyte invasion, reducing symptomatic forms in the human bloodstream, and blocking transmission to mosquitoes^5^. The first vaccine for malaria approved for use in select African countries by the WHO is the RTS,S/AS01 subunit vaccine, which aims to disrupt the malaria life cycle at the sporozoite stage prior to liver infection. *Plasmodium falciparum* sporozoites have a surface coating of circumsporozoite protein (*PfCSP*), which consists of amino-terminal and carboxyl-terminal domains flanking a central region of highly conserved NANP and NANP-like motifs repeated approximately 37–70 times depending on the strain. *PfCSP* is anchored to the sporozoite surface by myristoylated glycosylphosphatidylinositol (GPI) glycolipids, a post-translational replacement of a C-terminal hydrophobic tail^6^. RTS,S consists of 19 NANP major repeats and the C-terminal region displayed on a virus-like particle with hepatitis B surface antigen. Clinical trials with the RTS,S/AS01 vaccine showed variable levels of efficacy against infection that waned over time^7–9^. The second malaria vaccine to be approved by the WHO is the R21/Matrix-M vaccine that displays the same *PfCSP* antigenic regions as RTS,S; R21 was found to reach 75% efficacy against infection in African children, surpassing the efficacy of RTS,S^10^. Clinical trials studying vaccination with radiation-attenuated whole sporozoites have shown lower vaccine efficacy against natural *Plasmodium falciparum* infection compared to subunit vaccine counterparts in children and adults^11–13^. The mechanism of vaccine-induced antibody-mediated protection is still unclear, although some studies have shown sporozoite inhibition through Fc-dependent effector cell recruitment^14^, complement activation^15^, and other humoral mechanisms^16^. While previous studies dissected antibody responses to full-length *PfCSP* to determine the relative protection conferred by antibodies to each epitope region^17^, we hypothesized that immunodominant epitopes might be suppressing responses to other domains. Therefore, we tested each epitope region independently.

Recent studies have revealed that potent anti-*PfCSP* antibodies induced by sporozoite immunization bind to minor repeats of NPDP and NVDP found at the junction of the N-terminal domain and the central NANP-repeat region, and these minor repeats are termed junctional epitopes^18^. In controlled human malaria infection trials, passive administration of monoclonal antibodies (mAbs) that bind strongly to junctional epitopes was found to provide protection against malaria^19–22^. Several groups have made multimeric vaccine candidates targeting these junctional epitopes that show protection in challenge mouse models^23–25^, highlighting the potential importance of these epitopes despite their absence in RTS,S.

Other studies have highlighted the C-terminus as a significant contributor to protection in RTS,S^26,27^. The C-terminus, which has been shown to mediate hepatocyte invasion^28^, was found to fold into a structure termed the α-thrombospondin repeat (αTSR) domain^29^. The first structural study to explore anti-αTSR domain antibodies found that the nonprotective mAb 1710 utilized its CDRH3 to insert into a hydrophobic pocket after contacting the alpha-helix on one face of the molecule^30^. A recent study resolved the *PfCSP* C-terminus into two non-competing epitope regions: the strain-specific “alpha” epitopes targeted by 1710-like mAbs and the more conserved “beta” epitopes on the opposite side of the domain targeted by several new high-affinity mAbs^31^. Another study found that RTS,S was significantly less effective against malaria strains that utilize different C-terminus *PfCSP* sequences than the vaccine strain^32^. The observed reduction in efficacy against infection from diverse strains may be due to length polymorphism, as the number of NANP motifs ranges from 37 to 44 in different strains, but could also be attributed to sequence polymorphisms found in the C-terminus alpha epitope^33^. Therefore, we set out to further evaluate this C-terminus domain to find motifs that may elicit protective antibodies regardless of strain polymorphisms.

At the other terminus of *PfCSP*, the N-terminal domain is used by sporozoites during host cell traversal to mask the C-terminal domain^28^. Antibody responses to this domain have only recently been explored. 5D5 is a nonprotective murine antibody with high affinity to the N-terminal domain^34^. MAD2-6 is a human antibody that has protective function when presented as IgA but not as IgG^35^. Both target the same region of the N-terminal domain, raising the question of whether other N-terminal epitopes are immunogenic and potentially protective.

In the past decade, major advancements in mRNA delivery technology have resulted in the development of mRNA preclinical and clinical vaccines with favorable immunogenicity and safety profiles^36^. The nucleoside-modified mRNA immunogens delivered with lipid nanoparticles (LNPs) used by Pfizer/BioNTech and Moderna in SARS-CoV-2 vaccines have been proven to be safe and highly effective^37–39^. mRNA delivery is a newcomer to the field of malaria vaccinology, with only a few but promising studies targeting liver-resident memory T-cells^40^ or malaria antigens *PfCSP*, Pfs25, and PfRH5^41–43^. Thus, additional studies of mRNA delivery of malaria antigens in comparison to protein delivery are needed to identify potential next-generation malaria vaccines.

Here, we displayed potentially protective epitopes of *PfCSP* on two different glycosylated nanoparticles, ferritin (24mer) and lumazine synthase (60mer), for protein delivery. To assess the effectiveness of these immunogens, we immunized C57BL/6 J mice and evaluated the reduction in liver burden against live sporozoite challenge, which has predictive correlation with human protection^19,44^. These nanoparticles displayed one or more of the following: peptide (NANP)_6_, peptides covering the junctional region, wild-type CSP C-terminus, a C-terminal domain variant that was modified to abrogate alpha-site mAb binding without impacting binding to the novel beta-site epitope, and novel N-terminal epitopes targeted by antibodies elicited by KLH-N-terminal peptide mouse immunizations^45^. We selected a construct bringing together the most protective epitopes, namely a peptide covering the junctional region with additional NANPs, for delivery by mRNA using several different construct presentations, including two nanoparticle systems and two membrane-tethered systems: GPI-anchoring and a vesicular stomatitis virus glycoprotein G transmembrane (TM) domain. Furthermore, we evaluated *PfCSP* epitopes on immunogenic platforms in an unbiased manner to downselect the most protective epitopes and found, using mRNA delivery, promising sterilizing immunogens suggesting prospective platforms for future development.

## Results

### Immunogen design

**Fig. 1:**
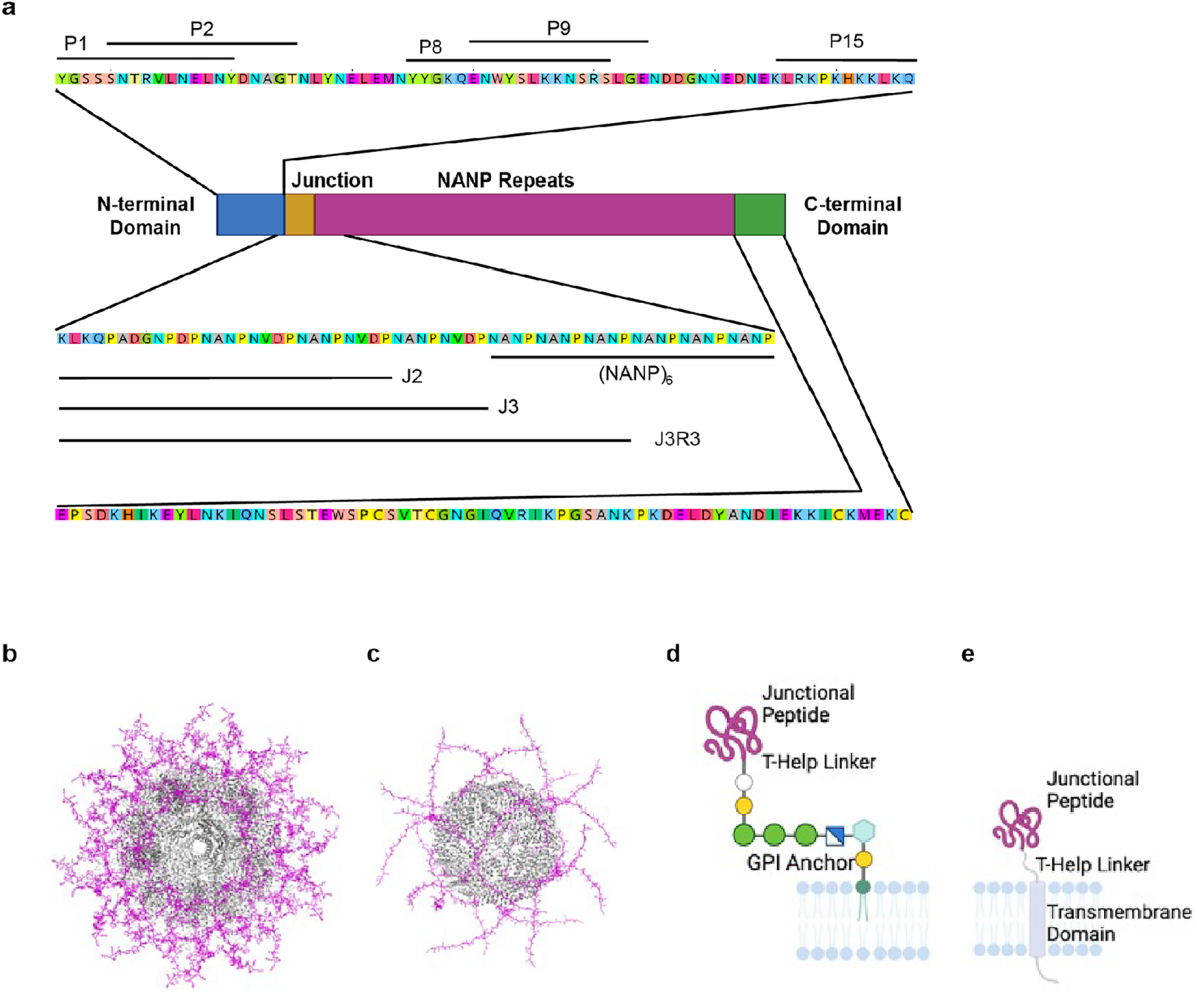
Circumsporozoite Epitope Regions. **a** N-terminal domain divided into five linear epitopes, termed P1, P2, P8, P9, and P15. Repeat epitopes divided into four peptides, J2, J3, J3R3, and (NANP)_6_. C-terminal domain sequence shown. **b** Model of J3R3 (magenta) displayed on 60mer lumazine synthase nanoparticle (grey). **c** Model of J3R3 (magenta) displayed on 24mer ferritin nanoparticle (grey). **d** Schematic of junctional peptide (magenta) displayed on GPI anchor with T-help linker. **e** Schematic of junctional peptide (magenta) displayed on transmembrane domain with T-help linker.

To develop immunogenic platforms for different *PfCSP* epitopes, we constructed ferritin- and lumazine synthase-based self-assembling nanoparticles displaying one or more different epitopes (Fig. 1b, c). We modified *H. pylori* ferritin (PDB:3bve) to engineer additional glycosylation sites. We also engineered additional glycosylation sites on *A. aeolicus* lumazine synthase (PDB:1hqk), a construct that had been previously modified to remove an unpaired cysteine and a buried glycosylation site^46^, add disulfide bridges, and resurface the active site^47^. The newly added glycosylation sites provide several potential advantages: (i) masking of non-CSP surfaces might reduce off-target responses; (ii) additional glycans might lead to improved in vivo trafficking to lymph nodes to augment immune responses^48^; and (iii) additional glycan allows for purification of nanoparticle immunogens without the use of potentially immunogenic affinity tags. In addition, we developed membrane-tethered platforms utilizing the VSV TM^49^ or the GPI signal sequence (Accession No. AL844502) natively used in *PfCSP*, to display junctional repeat peptides delivered by genetic immunization (Fig. 1d, e). We hypothesized that the membrane-tethered platforms would lack T-help in comparison to the nanoparticle platforms, and therefore we incorporated exogenous CD4 + T-cell epitopes, including PADRE^50^ and others from lumazine synthase^51^, into the linker.

We constructed immunogens displaying subunits from *PfCSP* strain 3D7 (Accession No. AL844502), the strain used in RTS,S/AS01, as shown in Fig. 1a. First, we fused (NANP)_6_ as a 24-residue peptide to the N-terminus of the nanoparticle protomers as a control for the NANP epitope of RTS,S in the context of our nanoparticles; we matched the number of repeat motifs found in the junctional region (NANPNVDP)_3_ as a higher number of repeat motifs interfered with nanoparticle formation. We displayed three additional peptides on nanoparticles to encompass the junctional epitope with the sequences beginning with Region I, the cleavage site important for parasite invasion^28^. We constructed a minimal junction peptide termed J2, a full-length junctional peptide termed J3, and a peptide termed J3R3 that added three NANP repeats onto the junctional peptide to combine junctional and NANP repeat epitopes. To focus the C-terminal immune response to the conserved epitope region, we used Rosetta to design a modified αTSR termed MD1 that minimized binding of known strain-specific antibodies without impacting antibody binding to the conserved epitope (Supplemental Fig. 1). We displayed both wild-type (WT) αTSR and MD1 αTSR on nanoparticles. We did not include the C-terminal linker, because no protective antibodies have been identified for this region^52,53^. We were unable to express the full N-terminal domain on nanoparticles. Instead, novel linear epitopes were identified through binding of novel antibodies to overlapping peptide sequences^45^, and these peptides, termed P1, P2, P7, P8, and P15, were selected for nanoparticle display as a representative set of N-terminal domain immunogenic epitopes. Nanoparticle formation was confirmed by size exclusion chromatography (SEC), SEC with multi-angle light scattering (SEC-MALS), and negative stain electron microscopy (nsEM) (Supplemental Figs. 2–10). Prospective epitopes for mRNA development were displayed on membrane-bound platforms for FACS characterization before production.

### In vitro characterization

**Fig. 2:**
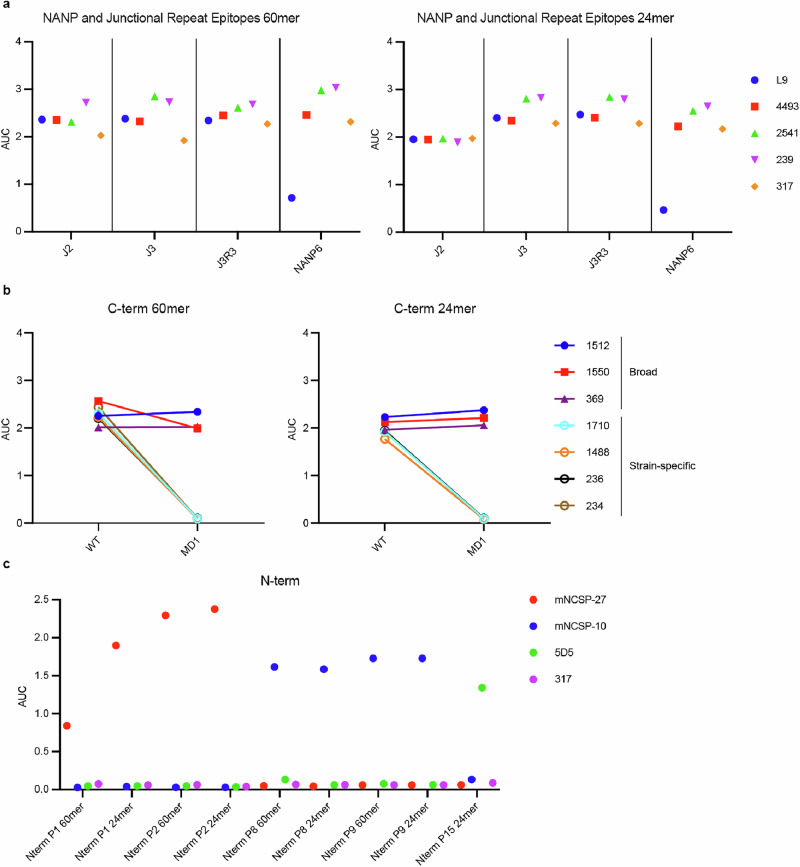
Nanoparticle Characterization by ELISA. **a** Binding, area under the curve (AUC), of anti-repeat antibodies 4493, 2541, 239, and 317 to directly coated repeat-displaying 24mer and 60mer immunogens. **b** Binding AUC of anti-C-terminal antibodies 1512, 1550, 1710, 1488, 236, and 234 to directly coated wild-type and modified-1 C-term displaying 24mer and 60mer immunogens. **c** Binding AUC of anti-N-terminal antibodies mNCSP-27, mNCSP-10, and 5D5 to directly coated N-term displaying 24mer and 60mer immunogens. Anti-repeat antibody 317 was used as a negative control.

We tested the nanoparticles for binding in ELISA against a select panel of mAbs. For the nanoparticles displaying junctional and NANP repeat epitopes, we confirmed antigenicity by testing against L9, a protective mAb targeting junctional repeat epitopes; 4493 and 2541, mAbs with cross reactivity for both junctional and NANP repeat epitopes^19^; and 239 and 317, mAbs initially found to target NANP repeat epitopes (Fig. 2a)^52,54^. We tested membrane-anchored immunogens displaying junctional and NANP repeat epitopes in FACS after transient DNA transfection against the same panel of mAbs (Supplemental Fig. 11) and found all immunogens bound these anti-repeat region antibodies. Related to the C-terminal αTSR domain, which contains conformational epitopes^53^, we confirmed appropriate epitope presentation following the modifications we had introduced by testing binding to conformation-dependent antibodies including several highly strain-specific antibodies (1710, 1488, 234, and 236) and two broadly cross-reactive antibodies (1512 and 1550) that bind a wide panel of αTSR domains from different strains (Fig. 2b)^31^. For the nanoparticles displaying N-terminal epitopes, we tested against a panel of mouse antibodies originally used to identify these peptides. P1 and P2 nanoparticles bound to mNCSP27. P8 and P9 nanoparticles bound to mNCSP10, and P15 nanoparticles bound to 5D5 (Fig. 2c). Thus, all immunogens were confirmed to display their respective epitopes.

### Serological response to vaccination

**Fig. 3:**
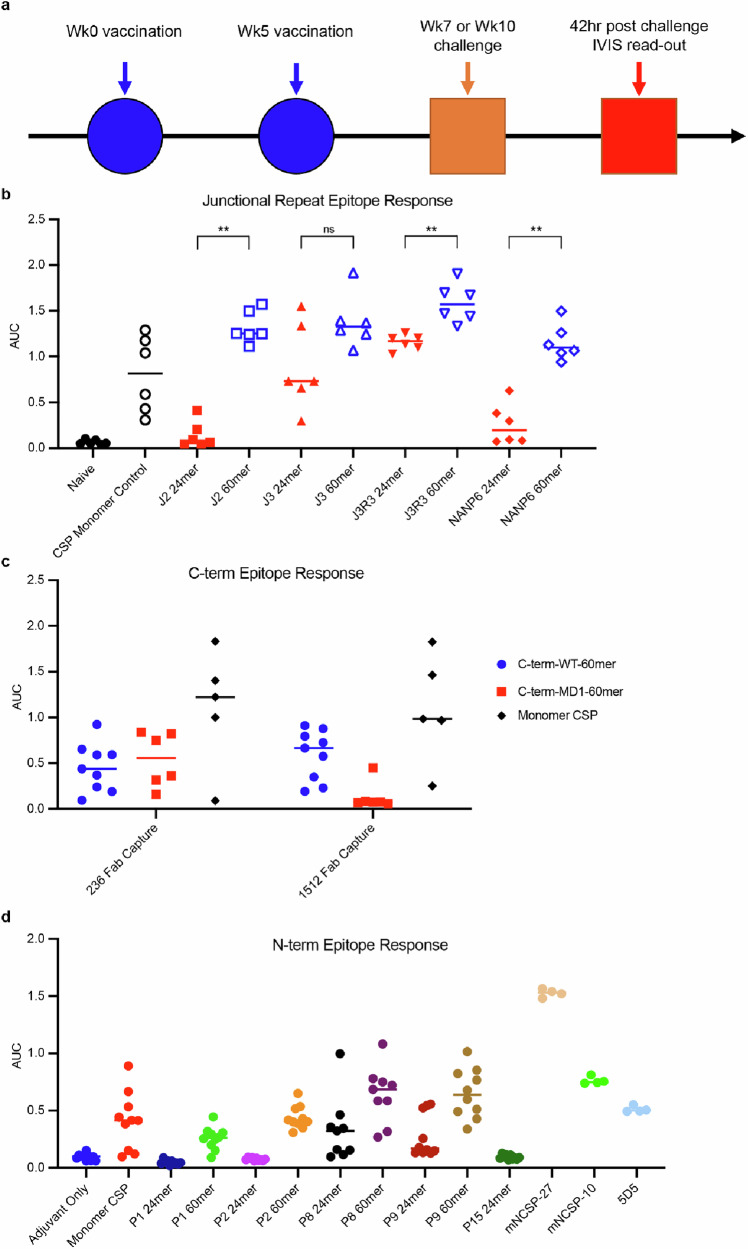
Serological response to vaccination by ELISA. **a** Schematic illustrating immunizations at weeks 0 and 5. Sera was taken from 1 week after last injection and challenge with live sporozoites was done at either 2 weeks or 5 weeks after last injection. Liver fluorescence was imaged at 42 h after challenge time. **b** Binding of sera to J2 peptide coated plates. Symbols represent area under the curve (AUC) values for individual mice (*n* = 6). When comparing serological responses between 24mers and 60mers displaying same peptides, significance testing was performed using a two-tailed Mann-Whitney U test; ns not significant; **p < 0.005. **c** Binding of sera to wild-type C-terminal domain captured on ELISA plates coated with either anti-alpha site antibody 236 or anti-beta site antibody 1512. Symbols represent AUC values for individual mice (*n* = 10); some groups have fewer than 10 data points due to insufficient sera. **d** Binding of sera to full-length N-terminal domain coated plates. Symbols represent AUC values for individual mice (*n* = 10). Groups were taken from different mouse trials.

To evaluate the immunogenicity of the nanoparticles, we vaccinated mice twice with proteins delivered with SMNP adjuvant^55^ (Fig. 3a). Because mouse age, strain, and sex can confound the raw fluorescence readouts in down-stream parasite challenge assays in naïve mice, we consistently utilized female C57BL/6 J mice that were 7–8 weeks old at the time of first immunization; C57BL/6 J mice are the most susceptible strain of mice to parasite infection and the most difficult mice to protect against challenge^56^. As a positive control immunogen, we used full-length monomeric *PfCSP* produced in *Lactococcus lactis*^57^, adjuvanted with SMNP.

We evaluated antibody binding responses using sera collected one week after the second vaccination (Week 5). For mice vaccinated with nanoparticles displaying peptides J2, J3, J3R3, and (NANP)_6_, we tested binding to a junctional peptide corresponding to J2. We found that 60mers elicited a stronger response than 24mers aside from J3 which merely trended higher (Fig. 3b). We also found that J3R3-60mer elicited the highest response to junctional epitopes compared to all immunogens targeting *PfCSP* repeats (Fig. 3b). For the C-terminal immunogens, we measured the serological response to the C-terminal αTSR domain captured on ELISA plates by either anti-alpha site antibody 236 or anti-beta site antibody 1512 thus binning the sera into either anti-alpha site response or anti-beta site response. We found that the WT αTSR immunogen elicited antibodies that bound to both alpha and beta C-terminal regions, while the MD1 αTSR immunogen elicited antibodies that bound to only the beta C-terminal region (Fig. 3c). To evaluate the effect of combined immunization with J3R3 and C-terminal immunogens, we compared the serum binding responses from mice immunized with single immunogens to those immunized with the combination. We found that the epitope-specific responses to the combination immunization were indistinguishable from immunization with the individual components (Supplemental Fig. 12a). Finally, we evaluated responses to P1, P2, P8, P9, and P15 immunogens except for P15 60mer, which did not form nanoparticles. We found P1 and P2 60mer but not 24mer sera showed detectable binding to full length N-terminal domain; all P8 and P9 sera showed detectable binding; and P15 24mer did not show detectable binding (Fig. 3d). For all protein candidates, we observed that repeat epitopes and C-terminal immunogens elicited stronger serological responses to their respective epitopes than N-terminal immunogens elicited for their respective epitopes (Fig. 3).

### Live parasite mouse challenge

**Fig. 4:**
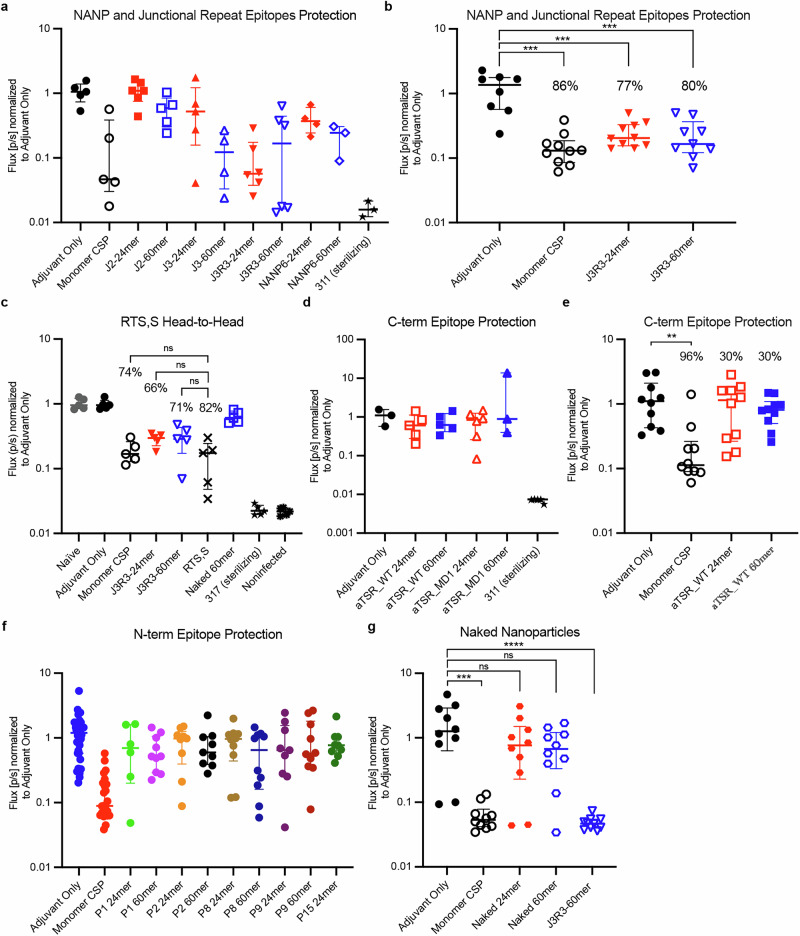
Mouse Protection assessed by Reduction of Parasite Liver Burden. Flux is normalized to adjuvant-only group. **a** Protection induced by nanoparticles (NANP)_6_ -24mer, (NANP)_6_ -60mer, peptide J2-24mer, peptide J2-60mer, peptide J3-24mer, peptide J3-60mer, J3R3-24mer, J3R3-24mer. Symbols represent individual mice (*n* = 6). **b** J3R3-24mer and J3R3-60mer were repeated in a larger immunization study with adjuvant-only control and monomer CSP. Symbols represent individual mice (*n* = 10). Protection was calculated as the reduction in signal compared to the adjuvant-only group. For comparisons to the adjuvant-only group, significance testing was performed using a two-tailed Mann-Whitney U test; ***, *p* < 0.0005. **c** Protection by J3R3 60mer tested head-to-head against RTS,S. Symbols represent individual mice (*n* = 5). Protection was calculated as the reduction in signal compared to the adjuvant-only group. Two-tailed Mann-Whitney U test p not less than 0.05 between reported immunogens and RTS,S/AS01. **d** Protection induced by wild-type and modified-1 C-terminal domains displayed on nanoparticles. Symbols represent individual mice (*n* = 6). **e** Wild-type C-terminal domain on 24mer or 60mer were repeated in a larger immunization study with adjuvant-only control and monomer CSP. Symbols represent individual mice (*n* = 10). Protection was calculated as the reduction in signal compared to the adjuvant-only group. To compare to adjuvant-only group, two-tailed Mann-Whitney U test was performed; ***, *p* < 0.0005. **f** Protection induced by nanoparticles displaying N-terminal epitopes P1-24mer, P1-60mer, P2-24mer, P2-60mer, P8-24mer, P8-60mer, P9-24mer, P9-60mer, and P15-24mer. Symbols represent individual mice (*n* = 10). **g** Protection induced by nanoparticles, 24mer and 60mer, displaying no malaria epitopes with adjuvant-only control and monomer CSP. Symbols represent individual mice (*n* = 10). To compare to adjuvant-only group, two-tailed Mann-Whitney U test was performed; ns not significant (*p* ≥ 0.05).

To measure the capabilities of the immunogens to reduce parasite load, at either 2 weeks or 5 weeks post boost, we performed intravenous high dose sporozoite challenges and monitored liver burden 42 hpost-challenge as described previously^56^. Challenge studies for repeat peptide and C-terminal epitopes were initially done with reduction in liver burden measured 5 weeks post boost with smaller group sizes, and the most promising immunogens were repeated in studies with larger group sizes with reduction in liver burden measured 2 weeks post boost (Fig. 4). All results were normalized to the adjuvant-only control group in each study, and reduction in liver burden signal for each immunogen was compared to the adjuvant only group of the same study since all groups were adjuvanted with SMNP aside from RTS,S. Where statistical significance was achieved, this was noted.

For all junctional and NANP repeat immunogens, we found that the 60mers tended to provide larger reduction in liver burden than the 24mers, consistent with the serological responses shown in Fig. 3. Immunization with (NANP)_6_ peptide 60mer provided 60% reduction in liver burden, and short junctional peptide (J2) 60mer provided 50% reduction in liver burden (Fig. 4a). The full-length junctional epitope (J3) 60mer demonstrated a larger reduction in liver burden at 80%, showing that this epitope contributes more than the traditionally studied NANP repeats. A 60mer combining J3 with three repeats of NANP (J3R3), mimicking the native composition of CSP, resulted in 90% reduction in liver burden (Fig. 4b). In a separate experiment, a head-to-head comparison of the best-performing immunogen J3R3-60mer/SMNP and RTS,S/AS01 was carried out in an independent laboratory. J3R3-60mer/SMNP showed comparable reduction in liver burden (median ± interquartile range, 71 ± 13%) to that for RTS,S/AS01 (82 ± 10%) and monomer CSP (83 ± 7%), despite having a substantially shorter epitope length (Fig. 4c).

In contrast, the C-terminal domain of CSP, whether wild-type or modified, provided a small reduction in liver burden, reaching a maximum of 36% (Fig. 4d, e). Combined immunization with C-term WT and J3R3 immunogens, approximating the possible combination of repeat and C-terminal epitopes on native CSP, showed reduction no higher than J3R3 alone (Supplemental Fig. 12b). Immunogens presenting peptides from the N-terminal domain, including peptides P1, P2, P8, P9, and P15, exhibited varying degrees of reduction in liver burden ranging from 27% to 60% (Fig. 4f).

Immunization with “naked” nanoparticles lacking malaria epitopes (CSP-negative nanoparticles) conferred nonsignificant levels of reduction in liver burden in this assay (Fig. 4g), suggesting that the base nanoparticle itself made minimal contributions to liver burden reduction for nanoparticles displaying CSP epitopes. However, despite the lack of significance, vaccination with CSP-negative nanoparticles trended toward lower flux levels compared to the adjuvant-only group, implying a potential impact on sporozoite fitness and liver invasion (Fig. 4g). This effect is unlikely to be driven by a humoral IgG response, as no circulating CSP-specific IgG titers were detected in these animals, as expected from the absence of CSP in the immunogen (Supplemental Fig. 13). All immunogens delivered as protein nanoparticles, despite varying levels in liver burden reduction, failed to provide sterilizing immunity, as all challenged mice, aside from those in groups receiving sterilizing mAbs, developed parasitemia. Therefore, we explored alternate methods of immunogen delivery.

### Immunization using mRNA-LNPs

**Fig. 5:**
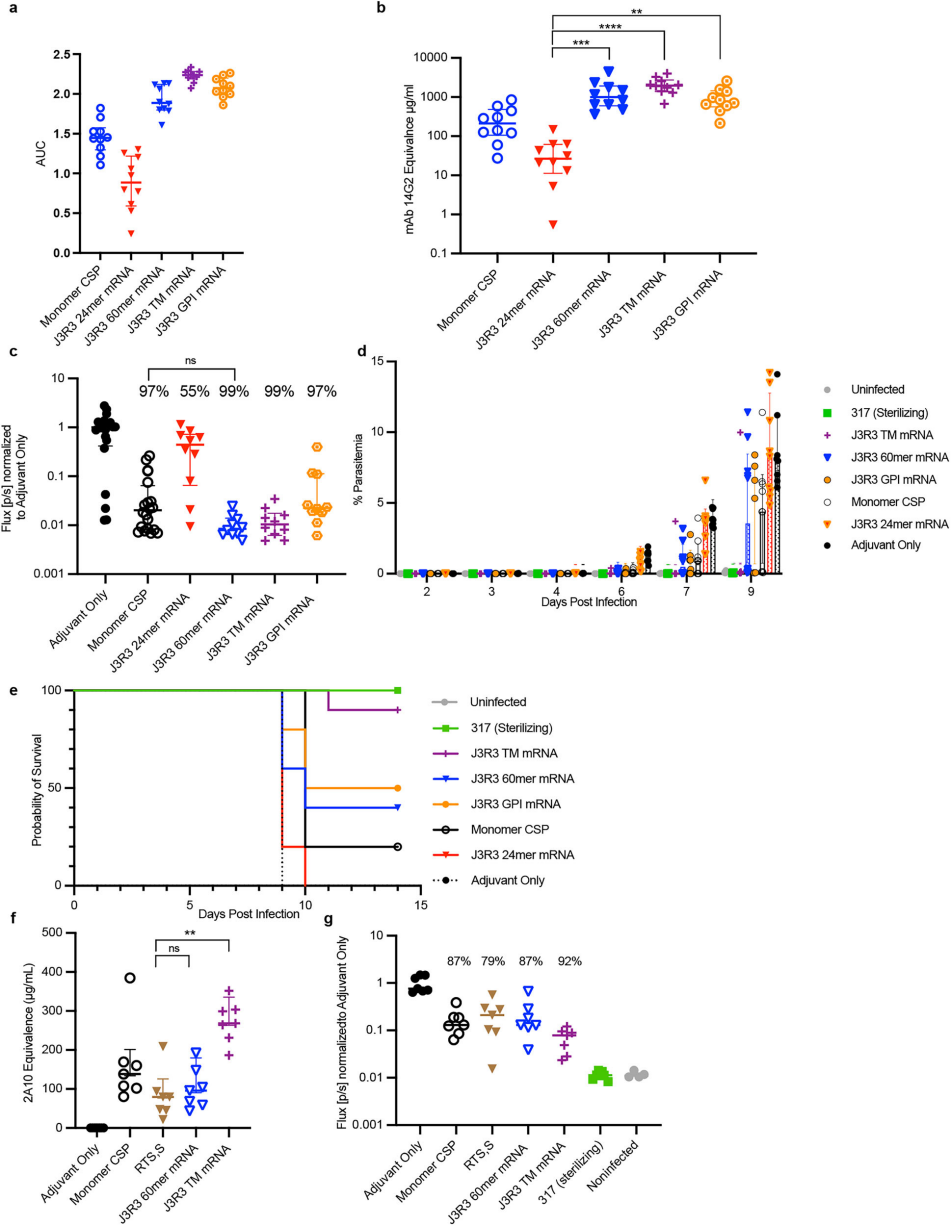
Protection by vaccination with mRNA-LNPs encoding different J3R3 antigens. **a** Binding of sera to J2 peptide-coated plates. Symbols represent area under the curve (AUC) values for individual mice (*n* = 10). **b** Circulating junctional specific IgG titers were interpolated using a 14G2 standard curve and sera from the indicated vaccination groups. Lines and error bars represent average mAb 14G2 equivalence and standard deviation respectively. Symbols represent individual mice per group (*n* = 10). To compare titers between groups, a Kruskal-Wallis test was performed with *p* < 0.05. **c** Protection by parasite burden liver assay of J3R3 on 24mer, 60mer, membrane-bound by VSV-TM, and membrane bound by GPI using mRNA-LNP. Symbols represent individual mice (*n* = 10). Protection was calculated as the reduction in signal compared to the adjuvant-only group. To compare to adjuvant-only group, two-tailed Mann-Whitney U test was performed with *p* < 0.05. **d** Percent composition of parasites in red blood cells in each vaccination group in a low parasite challenge model. Symbols represent individual mice (*n* = 10). Bar graph indicates median and interquartile range. **e** Mouse survival following parasite challenge after vaccination with the indicated mRNA-LNPs out of ten mice per group. **f** Circulating rCSP-specific IgG titers were interpolated using a 2A10 standard curve and sera from the indicated vaccination groups. Lines and error bars represent average mAb 14G2 equivalence and standard deviation respectively. To compare to the RTS,S group, a Kruskal-Wallis test was performed with *p* < 0.05. **g** Protection by J3R3 mRNA platforms was tested head-to-head against RTS,S. Symbols represent individual mice (*n* = 7). Protection was calculated as the reduction in signal compared to the adjuvant-only group. Two-tailed Mann-Whitney U test was performed with *p* < 0.05 between reported immunogens and RTS,S/AS01.

We employed mRNA-LNPs for the delivery of J3R3 nanoparticles (24mer and 60mer) and membrane-anchored J3R3 using both a GPI anchor and a VSV TM domain. J3R3 peptides were covalently linked to membrane tethers with GS linkers that included T helper epitopes, PADRE^58^ and two peptides from lumazine synthase^51^, to enhance immunogenicity. After immunizing mice with 10 µg of mRNA encoding these immunogens, we followed the same challenge schedule outlined in Fig. 3. At week six, animals were bled for serum antigen-specific IgG analysis. Titers were determined by interpolation using a NANP/junctional-specific murine mAb (14G2). mRNA-LNP delivery of J3R3-60mer, J3R3-TM, and J3R3-GPI resulted in average 14G2 equivalence values of 1408 µg/mL, 2096 µg/mL, and 999 µg/mL, respectively, all significantly higher than the J3R3-24mer mRNA group (Fig. 5a, b). For ease of comparison to previous protein immunogen studies, all results were normalized to the SMNP adjuvant-only control group in each study as before, and reduction in liver burden for each group was compared to reduction in liver burden for the SMNP adjuvant-only group of the same study. After a week seven challenge, we found that mRNA-LNP delivery of the J3R3-24mer elicited the smallest reduction in liver burden (56%), smaller than for protein delivery of J3R3-24mer and consistent with the low ELISA response to J3R3-24mer mRNA-LNP. J3R3-GPI mRNA-LNPs elicited reduction in liver burden of 97%. J3R3-60mer and J3R3-TM mRNA-LNPs both elicited the highest observed reduction in liver burden at 99% (Fig. 5c), with raw flux values (median ± interquartile range) 56088 ± 24888 photons/sec) and 69573 ± 38377, respectively. Furthermore, J3R3-60mer mRNA-LNPs elicited significantly higher reduction in liver burden than protein monomer CSP, the only immunogen tested in this study to do so (Fig. 5c).

We next immunized with protein monomer CSP, J3R3-24mer mRNA-LNP, J3R3-GPI mRNA-LNP, J3R3-60mer mRNA-LNPs, and J3R3-TM mRNA-LNP using the same schedule but with lower parasite challenge with 300 sporozoites. Small bleeds were taken from the mice following challenge, and red blood cells were monitored for parasitemia by flow cytometry (Fig. 5d, Supplemental Fig. 14). At 42 hours post infection, a majority of the immunogens exhibited levels of liver invasion inhibition like that of the mAb317 sterilizing control (Supplemental Fig. 15). At day three, mice in the adjuvant-only group begin to show parasitemia followed shortly by mice in other immunogen groups. At day six, all mice in the adjuvant-only group showed increased parasitemia, along with mice in all groups except those in the noninfected negative control group and the mAb 317-sterilized group. At day eight, all mice that showed parasitemia previously had increased parasite burden, while all mice that had shown no infection remained uninfected. At day nine, all mice in the adjuvant-only group exhibited sufficient malaria symptoms to require humane euthanasia as determined in the animal use protocol (Fig. 5e). Survival was monitored, and mice that exhibited no symptoms by day fourteen were considered to have to have received sterilizing protection from their respective immunogen (Fig. 5e). Mice passively immunized with 300 µg of mAb 317 remained parasite free and survived for the duration of the study (Fig. 5d, e). All mRNA-LNP immunogen groups except J3R3-TM showed significant drops in survival, despite a majority of the immunogens providing 97% or greater of inhibition of liver invasion (Fig. 5e).

We then tested lower doses (3 µg and 1 µg) of J3R3-60mer mRNA and J3R3-TM mRNA using the standard challenge model (Supplemental Fig. 16). 14G2-equivalent titers were measured from week six sera for each vaccine group. Increasing dosages of both 60mer mRNA and mRNA-TM resulted in corresponding increases in 14G2 equivalence, with maximum average titers of 2509 µg/mL and 2363 µg/mL, respectively (Supplemental Fig. 16a). Significant differences in liver burden reduction were observed between the 1 µg, 3 µg, and 10 µg groups, as evidenced by liver burden measurements and day six parasitemia levels (Supplemental Fig. 16b, c). Dose-dependent reduction in liver burden in the J3R3-60mer mRNA groups was most apparent in the survival analysis (Supplemental Fig. 16d). Overall, the trends observed in liver burden, parasitemia, and survival analyses suggested dose-dependent reductions in liver burden from mRNA-VSV-TM that peaked at the maximum tested dose of 10 µg. Dose-dependent liver burden reduction for J3R3-60mer was less clear, though the data suggested that doses higher than 1 µg confer increased effect.

Subsequently, mRNA-delivered J3R3-60mer and J3R3-TM were directly compared to RTS,S/AS01_E_ in an independent laboratory, using the same immunization schedule and a modified challenge protocol utilizing 2000 sporozoites per mouse. CSP-specific IgG titers were quantified by interpolation using the murine NANP-specific mAb 2A10 in an ELISA format. Both J3R3-60mer mRNA-LNP and J3R3-TM mRNA-LNP elicited higher NANP-specific IgG titers, with values of 101 µg/mL ± 53 and 272 µg/mL ± 53.4, respectively, compared to RTS,S, which averaged 82 µg/mL ± 61, but the difference was significant only for J3R3-TM mRNA-LNP (*p* = 0.0012) (Fig. 5f). Immunization with J3R3-TM mRNA-LNP, J3R3-60mer mRNA-LNP, or RTS,S resulted in inhibition of liver invasion, with similar median inhibition values of 92%, 87%, and 79%, respectively (Fig. 5g). Given the lower inhibition of liver invasion of RTS,S, it is unlikely that it would provide sterilizing protection and significant survival to these animals like we show with J3R3-TM mRNA-LNP in Fig. 5e. This is in line with what has been shown elsewhere with two doses of RTS,S at 5 µg per dose^59^.

## Discussion

This study initially evaluated multiple potentially protective *PfCSP* epitopes in an unbiased manner on two glycosylated nanoparticle platforms. We found that the most effective protein immunogen, J3R3-60mer, exhibited a reduction in liver burden on par with RTS,S and, when delivered by mRNA-LNPs, elicited near-sterilizing immunity in a mouse model with high-dose parasite challenge after two vaccinations. mRNA-LNP delivery of J3R3-24mer elicited less reduction in liver burden than protein, which might have been due to inefficient nanoparticle formation or low expression similar to another study using HIV-ferritin^60^. mRNA-LNP delivery of the transmembrane-anchored J3R3-TM also elicited near-sterilizing immunity similar to that of J3R3-60mer mRNA-LNP. J3R3-60mer mRNA-LNP showed lower vaccine dose dependence than J3R3-TM mRNA-LNP for decreasing parasite infection. Using parasite challenge after two-dose immunization, we tracked parasitemia and survival in the animals immunized with mRNA-LNP for J3R3-24mer, J3R3-GPI, J3R3-60mer, and J3R3-TM. Notably, J3R3-TM mRNA-LNP induced sterilizing immunity in a higher percentage of mice, as determined by parasitemia and survival, than J3R3-60mer mRNA-LNP despite an identical reduction in liver burden in the high-dose parasite challenge model. Although we were unable to formulate full-length *PfCSP* mRNA for these experiments, these results appeared to be superior to those for a previously reported mRNA vaccine using full-length *PfCSP*^42^, where protection was reported only after four immunizations.

Of the epitopes evaluated, we concluded that repeat epitopes were the most protective epitopes in *PfCSP*. The junctional repeat epitopes NPDP and NPNV combined with the NANP repeat epitope of RTS,S/AS01 (J3R3) were more effective for liver burden reduction than either alone (J3 and NANP_6_) and elicited equivalent reduction in liver burden as recombinant full-length monomeric CSP protein despite a much shorter repeat epitope length, (NXXP)_10_ compared to (NXXP)_43_. In contrast to our findings, a recent study (Krenger et al. ^61^) investigating similar epitopes presented on virus-like particles concluded that adding junctional epitopes to NANP repeat epitopes did not improve vaccine efficacy. Krenger et al. used (NANP)_19_ paired with peptides nearly identical to the J1 and J2 epitopes, presented on virus-like particles and administered without an adjuvant. However, Krenger et al. paired junctional epitopes with an overabundance of NANP repeats, which might have over-focused the immune response on NANP^62^. This, in turn, might have limited junctional-specific antibody titers and reduced the overall protective potency of the antibody response. A previous study using a *PfCSP*-based ferritin nanoparticle similar to J3R3-24mer reached a similar conclusion that limiting the NANP repeat valency may be important to inducing robust anti-junction response^62^. Additionally, another study showed that immunization with 9 NANP repeats elicited stronger protection than immunization with 27 NANP repeats^63^. Taking our study in context with these others, it seems that J3R3 represents an effective combination of junctional epitope repeats and NANP repeats, although it remains possible that a more optimal balance might be found. While J3R3-TM mRNA-LNP induced significantly higher circulating NANP-specific IgG titers compared to RTS,S at the doses tested, it did not confer significantly greater protection, confirming that anti-repeat antibody titers alone do not fully correlate with protection^64,65^. Although the differences in relative dose levels and types of adjuvants make any direct comparisons between mRNA and protein difficult, the potent protection elicited by most of the mRNA immunogens tested here suggests that further characterization and exploration are warranted. Thus, our study utilizing mRNA delivery, in addition to recent studies in the field probing junctional epitope responses, has illustrated that junctional peptide immunogens delivered by mRNA provide powerful protection in mouse models and could be considered as candidates in the next generation of repeat epitope-focused *PfCSP* vaccines.

Regarding non-repeat region epitopes, we found that neither the C-terminal nor the N-terminal epitopes induced substantial protective responses in the mouse models employed here, despite being immunogenic. Our results corroborated a recent study showing that 73 C-terminus-specific antibodies elicited in malaria-naïve individuals immunized with attenuated sporozoites were lacking in anti-parasite activity^53^. In addition, cocktail immunization with the C-terminal domain combined with our best junctional immunogen did not show additive or synergistic levels of protection compared to the junctional immunogen alone, despite the elicitation of anti-C-terminal antibodies. This was consistent with prior research that found that the combined transfer of anti-C-terminal antibodies and anti-repeat antibodies did not yield a higher level of protection when compared to anti-repeat antibodies alone^66^. These findings underscore the challenge faced by anti-C-terminal antibodies in accessing their epitopes, which are situated proximal to the parasite surface and are surrounded by a dense coat of repeat epitopes. It is known that anti-N-terminal antibodies are poorly elicited in mouse and rabbit animal models by full-length CSP^67^. A recent study immunized mice with the N-terminal domain fused to KLH and found anti-CSP antibodies to novel linear epitopes in the N-terminal domain^45^. We found that while these linear epitopes elicited serological responses, none of the tested peptides elicited strong protective responses. Our data do not provide evidence to support inclusion of N-terminal and C-terminal epitopes in a malaria vaccine, but our studies do not rule out the possibility that different immunogens eliciting responses to these epitopes might influence the sporozoite’s ability to invade hepatocytes.

In this study, we restricted immunizations to female C57BL/6 J mice that were 7-8 weeks old at the time of the first immunization, to avoid the confounding factors of age, strain, and sex that impact the raw signals in liver burden fluorescence. It is important to note that significant reduction in liver burden in this assay does not necessarily mean sterile protection. However, the liver burden assay has been shown to successfully distinguish between protective and non-protective antibodies passively infused in mouse studies that are age-matched, strain-matched, or sex-matched^56^. Although C57BL/6 J mice do not possess IgG subclass 2a genes, and there are some suggestions that IgG subclasses can play a role in protection, there is no direct evidence that specific IgG subclasses can regulate anti-parasite activity^68^. It has been shown that the same antibodies that show high protection in the liver burden assay in C57BL/6 J mice are protective in humans^19,22,69^. Most importantly, to demonstrate the relevance of this mouse model to humans, this liver burden assay using C57BL/6 J female mice has been shown to be capable of distinguishing protected and non-protected human plasma samples from a controlled human malaria infection study^59^.

Our study had several limitations. Responses to mRNA/LNP delivered immunogens are affected by differing efficiencies of protein expression, nanoparticle formation, or cell surface antigen display that make interpretation of differences compared to protein immunogens difficult; such effects are also difficult to distinguish from effects on immunogenicity due to different immunogen formats, such as nanoparticle versus transmembrane-anchored peptide. We did not attempt to delineate the factors contributing to immunogenicity differences between protein and mRNA immunogens or between different mRNA immunogens. The liver burden assay gauges protection at forty-two hours post-challenge, a time point unsuitable to assess vaccine durability. Future studies to evaluate durability in mice could be conducted by challenging months after the last boost. However, we also note that mouse models are not well-suited for assessing durability—such studies would probably be more informative if conducted in non-human primates or humans. The fact that N-terminal domain epitopes did not confer strong protection in the mouse model employed may be a limitation of the parasite intravenous challenge route, as the N-terminal domain plays a role in dermal traversal following mosquito bite^28^. Future studies could also test these immunogens using mosquito bite challenge, although we note that the parasite liver burden assay and mosquito bite challenge results correlate well when evaluating anti-repeat epitope antibodies^70^. Although it has been shown that the skin is a key site for sporozoite inhibition^71^, our studies utilizing IV challenge were not sensitive to this aspect of potential protection. Therefore, we would like to emphasize that the high levels of inhibition of liver invasion and protection provided by these immunogens with the IV challenge method should be seen as a high benchmark. Our findings demonstrate that, even when bypassing a key site of parasite inhibition in the skin, these immunogens are still capable of eliciting a protective immune response elsewhere in the animal. We hypothesize that, based on our results in the IV challenge model, a mosquito bite model would confirm the potency of our top immunogen. Additionally, we believe it could offer further insights into the mechanisms of protection elicited by both our highly potent and less potent immunogens and therefore should be pursued in follow-up studies. Overall, the in vivo model we used may not have the capability to accurately determine the inhibition percentage of moderately protective immunogens against high-dose parasite challenge. The model appears to be robust at identifying strong protectors, as demonstrated by our J3R3 immunogens, RTS,S, and other repeat-region mAbs evaluated previously^18,54,72–74^. However, measuring protective efficacy at low-to-moderate levels of protection would likely require lower parasite dose challenges or significantly larger group sizes^31,53^. Although our experiments were not explicitly repeated in a traditional replicate format, we demonstrate across multiple independent studies that identical immunogens and doses consistently produce reproducible levels of inhibition of liver invasion. This consistency is particularly evident with monomer CSP (Figs. 4a, b, e, f, g, 5c, S12b, S16b), J3R3-60mer (Fig. 4b, c), J3R3-60mer mRNA (Figs. 5c, g, S16b), and J3R3-TM mRNA (Figs. 5c, g, S16b). However, given our use of high parasite loads during challenge, sterilizing protection for our top immunogens was less consistent. For example, J3R3-TM mRNA prevented parasitemia and allowed for survival in 90% of animals in Fig. 5, but only 50% in Supplemental Fig. 16. Despite this variability, we believe the ability to achieve at least 50% protection and up to 90% protection under these stringent conditions and with only a two-dose vaccination regimen represents a favorable and promising outcome. Finally, we are limited in the functional characterization of elicited antibodies such as Fc-dependent effector cell recruitment, as it is not known how these humoral responses in wild-type mice translate to findings in vaccinated humans. Thus, future studies could also include functional antibody analysis in non-human primates.

How might this study contribute to improved malaria vaccines? Our best performing mRNA immunogens elicited near-sterilizing immunity in a mouse model after two vaccinations, but their potential for similar human protection is uncertain. With the context that mRNA delivery enables rapid translation of promising vaccines to human clinical testing, and having acknowledged that a further mouse mosquito bite challenge study might be useful to confirm the potency of our best immunogens, we believe that controlled human challenge studies with the *PfCSP* mRNA vaccines reported here should be considered. Such studies would provide further insight into the predictive capacity of the mouse challenge model and evaluate the degree to which these or similar vaccine candidates might contribute to a next-generation malaria vaccine. A previous study has shown that both neutralizing antibodies and CD8+ cells are required for sterile immunity to sporozoite challenge^75^. In addition, another study showed that a cocktail of mRNA delivered immunogens of different epitopes does not interfere with individual epitope responses^42^, paving the way for a malaria cocktail vaccine that can potentially elicit potent protection to multiple stages by B-cell stimulation to target blood-stage, sexual-stage, and liver-stage antigens as well as stimulation of liver-resident memory T-cells^40^. Our most protective mRNA *PfCSP* subunit immunogens have the potential to contribute to a next-generation mRNA malaria cocktail vaccine capable of inducing sterilizing immunity.

## Methods

### DNA gene synthesis and cloning

All genes were synthesized at Genscript, Inc. Nanoparticles were cloned into pHLsec between the leader and a double stop coding using the AgeI and KpnI cloning sites. Antibody heavy chains were cloned into pCW-CHIg-hG1 between the leader and IgG1 human constant domain using EcoRI and NheI cloning sites. Double stop codons were inserted after the CH_1_ region for Fab production. Kappa chains were cloned into pCW-CLIg-hk between the leader and human kappa constant region using the EcoRI and BsiWI. Lambda chains were cloned into pCW-CLIg-hL2 between the leader sequence and the human lambda constant region using the EcoRI and AvrII cloning sites.

We made immunogens displayed on modified self-assembling nanoparticles ferritin and lumazine synthase. We modified *H. pylori* ferritin (PDB:3bve) to remove a problematic naturally occurring glycosylation site at position 18 and engineered four additional glycosylation sites introduced at positions 68, 78, 95, and 102. Peptides were linked directly without a GGS linker to utilize proline in orienting the peptide away from the particle. We modified *A. aeolicus* lumazine synthase (PDB:1hqk) to mutate away an unpaired cysteine at position 37 and a buried glycosylation site at position 102. Four cysteines were introduced to design two extra disulfide bridges. Three mutations were introduced to knock out the active sites of the nanoparticle. And finally, one extra glycosylation site was introduced at position 71 and two glycosylation sites were added to the C-terminus.

### Immunogen amino acid sequences

#### (NANP)_6_ 24mer

NANPNANPNANPNANPNANPNANPLSKDIIKLLNEQVNKEMQSSNLYMSMSSWCYTHSLDGAGLFLFDHAAEEYEHAKKLIIFLNENNVPVNLTSISAPEHNFTGLTQIFQKAYEHEQNISESINNITDHAIKSKDHATFNFLQWYVAEQHEEEVLFKDILDKIELIGNENHGLYLADQYVKGIAKSRKS

#### (NANP)_6_ 60mer

NANPNANPNANPNANPNANPNANPGSGNGTGGSMQIYEGKLTAEGLRFGIVASRANHALVDRLVEGAIDAIVRHGGREEDITLVRVCGSWEIPVAAGELARKENISAVIAIGVLCRGATPSFDYIASEVSKGLADLSLELRKPITFGVITADTLEQAIEAAGTCHGNKGWEAALCAIEMANLFKSLRGGSNGTGGSGGSNGT

#### J2 24mer

KLKQPADGNPDPNANPNVDPNANPNVDPLSKDIIKLLNEQVNKEMQSSNLYMSMSSWCYTHSLDGAGLFLFDHAAEEYEHAKKLIIFLNENNVPVNLTSISAPEHNFTGLTQIFQKAYEHEQNISESINNITDHAIKSKDHATFNFLQWYVAEQHEEEVLFKDILDKIELIGNENHGLYLADQYVKGIAKSRKS

#### J2 60mer

KLKQPADGNPDPNANPNVDPNANPNVDPGSGNGTGGSMQIYEGKLTAEGLRFGIVASRANHALVDRLVEGAIDAIVRHGGREEDITLVRVCGSWEIPVAAGELARKENISAVIAIGVLCRGATPSFDYIASEVSKGLADLSLELRKPITFGVITADTLEQAIEAAGTCHGNKGWEAALCAIEMANLFKSLRGGSNGTGGSGGSNGT

#### J3 24mer

KLKQPADGNPDPNANPNVDPNANPNVDPNANPNVDPLSKDIIKLLNEQVNKEMQSSNLYMSMSSWCYTHSLDGAGLFLFDHAAEEYEHAKKLIIFLNENNVPVNLTSISAPEHNFTGLTQIFQKAYEHEQNISESINNITDHAIKSKDHATFNFLQWYVAEQHEEEVLFKDILDKIELIGNENHGLYLADQYVKGIAKSRKS

#### J3 60mer

KLKQPADGNPDPNANPNVDPNANPNVDPNANPNVDPGSGNGTGGSMQIYEGKLTAEGLRFGIVASRANHALVDRLVEGAIDAIVRHGGREEDITLVRVCGSWEIPVAAGELARKENISAVIAIGVLCRGATPSFDYIASEVSKGLADLSLELRKPITFGVITADTLEQAIEAAGTCHGNKGWEAALCAIEMANLFKSLRGGSNGTGGSGGSNGT

#### J3R3 24mer

KLKQPADGNPDPNANPNVDPNANPNVDPNANPNVDPNANPNANPNANPLSKDIIKLLNEQVNKEMQSSNLYMSMSSWCYTHSLDGAGLFLFDHAAEEYEHAKKLIIFLNENNVPVNLTSISAPEHNFTGLTQIFQKAYEHEQNISESINNITDHAIKSKDHATFNFLQWYVAEQHEEEVLFKDILDKIELIGNENHGLYLADQYVKGIAKSRKS

#### J3R3 60mer

KLKQPADGNPDPNANPNVDPNANPNVDPNANPNVDPNANPNANPNANPGSGNGTGGSMQIYEGKLTAEGLRFGIVASRANHALVDRLVEGAIDAIVRHGGREEDITLVRVCGSWEIPVAAGELARKENISAVIAIGVLCRGATPSFDYIASEVSKGLADLSLELRKPITFGVITADTLEQAIEAAGTCHGNKGWEAALCAIEMANLFKSLRGGSNGTGGSGGSNGT

#### C-term WT 24mer

EPSDKHIKEYLNKIQNSLSTEWSPCSVTCGNGIQVRIKPGSANKPKDELDYANDIEKKICKMEKCGGSLSKDIIKLLNEQVNKEMQSSNLYMSMSSWCYTHSLDGAGLFLFDHAAEEYEHAKKLIIFLNENNVPVNLTSISAPEHNFTGLTQIFQKAYEHEQNISESINNITDHAIKSKDHATFNFLQWYVAEQHEEEVLFKDILDKIELIGNENHGLYLADQYVKGIAKSRKS

#### C-term WT 60mer

EPSDKHIKEYLNKIQNSLSTEWSPCSVTCGNGIQVRIKPGSANKPKDELDYANDIEKKICKMEKCGGSMQIYEGKLTAEGLRFGIVASRANHALVDRLVEGAIDAIVRHGGREEDITLVRVCGSWEIPVAAGELARKENISAVIAIGVLCRGATPSFDYIASEVSKGLADLSLELRKPITFGVITADTLEQAIEAAGTCHGNKGWEAALCAIEMANLFKSLRGGSNGTGGSGGSNGT

#### C-term MD1 24mer

EPSDNHTKSYYAKIQNSLSTEWSPCSVTCGNGIQVRIKPGSANKPKANLTYANDIEKKICKMEKCGGSLSKDIIKLLNEQVNKEMQSSNLYMSMSSWCYTHSLDGAGLFLFDHAAEEYEHAKKLIIFLNENNVPVNLTSISAPEHNFTGLTQIFQKAYEHEQNISESINNITDHAIKSKDHATFNFLQWYVAEQHEEEVLFKDILDKIELIGNENHGLYLADQYVKGIAKSRKS

#### C-term MD1 60mer

EPSDNHTKSYYAKIQNSLSTEWSPCSVTCGNGIQVRIKPGSANKPKANLTYANDIEKKICKMEKCGGSMQIYEGKLTAEGLRFGIVASRANHALVDRLVEGAIDAIVRHGGREEDITLVRVCGSWEIPVAAGELARKENISAVIAIGVLCRGATPSFDYIASEVSKGLADLSLELRKPITFGVITADTLEQAIEAAGTCHGNKGWEAALCAIEMANLFKSLRGGSNGTGGSGGSNGT

#### P1 24mer

YGSSSNTRVLNELNYLSKDIIKLLNEQVNKEMQSSNLYMSMSSWCYTHSLDGAGLFLFDHAAEEYEHAKKLIIFLNENNVPVNLTSISAPEHNFTGLTQIFQKAYEHEQNISESINNITDHAIKSKDHATFNFLQWYVAEQHEEEVLFKDILDKIELIGNENHGLYLADQYVKGIAKSRKS

#### P1 60mer

YGSSSNTRVLNELNYGSGNGTGGSMQIYEGKLTAEGLRFGIVASRANHALVDRLVEGAIDAIVRHGGREEDITLVRVCGSWEIPVAAGELARKENISAVIAIGVLCRGATPSFDYIASEVSKGLADLSLELRKPITFGVITADTLEQAIEAAGTCHGNKGWEAALCAIEMANLFKSLRGGSNGTGGSGGSNGT

#### P2 24mer

SNTRVLNELNYDNAGLSKDIIKLLNEQVNKEMQSSNLYMSMSSWCYTHSLDGAGLFLFDHAAEEYEHAKKLIIFLNENNVPVNLTSISAPEHNFTGLTQIFQKAYEHEQNISESINNITDHAIKSKDHATFNFLQWYVAEQHEEEVLFKDILDKIELIGNENHGLYLADQYVKGIAKSRKS

#### P2 60mer

SNTRVLNELNYDNAGGSGNGTGGSMQIYEGKLTAEGLRFGIVASRANHALVDRLVEGAIDAIVRHGGREEDITLVRVCGSWEIPVAAGELARKENISAVIAIGVLCRGATPSFDYIASEVSKGLADLSLELRKPITFGVITADTLEQAIEAAGTCHGNKGWEAALCAIEMANLFKSLRGGSNGTGGSGGSNGT

#### P8 24mer

NYYGKQENWYSLKKNLSKDIIKLLNEQVNKEMQSSNLYMSMSSWCYTHSLDGAGLFLFDHAAEEYEHAKKLIIFLNENNVPVNLTSISAPEHNFTGLTQIFQKAYEHEQNISESINNITDHAIKSKDHATFNFLQWYVAEQHEEEVLFKDILDKIELIGNENHGLYLADQYVKGIAKSRKS

#### P8 60mer

NYYGKQENWYSLKKNGSGNGTGGSMQIYEGKLTAEGLRFGIVASRANHALVDRLVEGAIDAIVRHGGREEDITLVRVCGSWEIPVAAGELARKENISAVIAIGVLCRGATPSFDYIASEVSKGLADLSLELRKPITFGVITADTLEQAIEAAGTCHGNKGWEAALCAIEMANLFKSLRGGSNGTGGSGGSNGT

#### P9 24mer

KQENWYSLKKNSRSLLSKDIIKLLNEQVNKEMQSSNLYMSMSSWCYTHSLDGAGLFLFDHAAEEYEHAKKLIIFLNENNVPVNLTSISAPEHNFTGLTQIFQKAYEHEQNISESINNITDHAIKSKDHATFNFLQWYVAEQHEEEVLFKDILDKIELIGNENHGLYLADQYVKGIAKSRKS

#### P9 60mer

KQENWYSLKKNSRSLGSGNGTGGSMQIYEGKLTAEGLRFGIVASRANHALVDRLVEGAIDAIVRHGGREEDITLVRVCGSWEIPVAAGELARKENISAVIAIGVLCRGATPSFDYIASEVSKGLADLSLELRKPITFGVITADTLEQAIEAAGTCHGNKGWEAALCAIEMANLFKSLRGGSNGTGGSGGSNGT

#### P15 24mer

DNEKLRKPKHKKLKQLSKDIIKLLNEQVNKEMQSSNLYMSMSSWCYTHSLDGAGLFLFDHAAEEYEHAKKLIIFLNENNVPVNLTSISAPEHNFTGLTQIFQKAYEHEQNISESINNITDHAIKSKDHATFNFLQWYVAEQHEEEVLFKDILDKIELIGNENHGLYLADQYVKGIAKSRKS

#### J3R3 LS-PADRE GPI (referred to as J3R3 GPI)

KLKQPADGNPDPNANPNVDPNANPNVDPNANPNVDPNANPNANPNANPGGSATPHFDYIASEVSKGLADLGGSFGVITADTLEQAIERGGSAKFVAAWTLKAAAGGSSSVFNVVNSSIGLIMVLSFLFLN

#### J3R3 LS-PADRE VSV-TM (referred to as J3R3-TM)

KLKQPADGNPDPNANPNVDPNANPNVDPNANPNVDPNANPNANPNANPGGSATPHFDYIASEVSKGLADLGGSFGVITADTLEQAIERGGSAKFVAAWTLKAAAGGSSKSSIASFFFIIGLIIGLFLVLR

### Protein purification

DNA was transfected with a plasmid into FreeStyle 293 F cells (Invitrogen, Cat no. R79007) using 293Fectin (ThermoFisher), and proteins were expressed at 37 ˚C for four days. NPs were purified using snowdrop lectin-conjugated agarose beads (Vector laboratories), followed by gel-filtration using a Superose 6 size-exclusion chromatography column (GE Healthcare). NP-assembly was assessed by SEC + multiangle light scattering (SEC-MALS) using a Superose 6 10/300 column (GE Healthcare) at a flow rate of 0.5 ml/min followed by DAWN HELEOS II and Optilab T-rEX detectors (Wyatt Technology), correcting for the glycan molecular mass by applying the built-in protein-conjugate analysis (ASTRA).

Full-length monomeric protein *PfCSP* was produced in *Lactococcus lactis* as described previously^57^.

### Fab and antibody purification

Paired HC and LC Fab variable region sequences were gene-synthesized and inserted into human Fab HC constant region expressing vector pFabCW and human lambda or kappa expressing vectors pCW-CLig-hL2 or pCW-CLig-hk, respectively. Fabs were expressed in 500 mL FreeStyle™ 293 F cell cultures or 30 mL ExpiCHO™ cell cultures (Thermo Fisher Scientific, Cat# A29133). For 293 F cell transfection, 300 μg of HC and 150 μg of LC plasmids were mixed with 225 μg polyethylenimine (PEI; 1:3 DNA:PEI ratio) in 5 mL of Opti-MEM™ reduced serum medium (Thermo Fisher Scientific, Cat# 31985070) for 30 min, then added to 293 F cells. Supernatant was collected after 5–6 days. ExpiCHO™ cell cultures were transfected according to manufacturer instructions, using 12.5 μg HC and 31.2 μg LC plasmids. Supernatant was collected 8 days post-transfection. Harvested supernatants were filtered through 0.45 or 0.25 μm membrane filters and batch bound to CaptureSelect CH1-XL Affinity resin (Thermo Fisher Scientific, Cat# 1943462005). Resin was washed with PBS, and captured Fabs were eluted with 50 mM NaOAc pH 4.0, buffer exchanged into 1× PBS, and concentrated using a 30k MWCO concentrator.

For expression of IgG the HC variable region was cloned into the pCW-CHIg-hG1 vector. Transfection was carried out as described above, but batch binding occurred overnight at 4 °C to Protein A resin (Thermo Fisher Scientific, Cat# 20334) while on a rocker. Unbound supernatant was allowed to flow through, and the resin was washed with PBS until protein A280 reading of the flowthrough measured by a nanodrop reached background levels. Protein A bound IgG was eluted with 0.1 M Glycine pH 2.7. Eluted mAbs were buffer exchanged into 1× PBS and concentrated using a 50 K MWCO concentrator (Millipore).

### Protein modeling and design with Rosetta

Analysis for possible glycosylation sites was done using GlycanTreeModeler as described previously^76^. Rosetta fixed backbone, relax, and scoring were used to prescreen core mutations to find suitable mutations.

### mRNA-LNP formulation

mRNA-LNPs for J3R3-60mer, J3R3-24mer, J3R3-TM, and J3R3-GPI were synthesized in vitro and passed quality control tests as previously described in ref. ^77^. The secretion signal sequence for human IL-2 (MYRMQLLSCIALSLALVTNS) was used for J3R3-TM and J3R3-GPI mRNA constructs, and the secretion signal sequence from the pHLsec vector (MGILPSPGMPALLSLVSLLSVLLMGCVAETG)^78^ was used for J3R3-60mer and J3R3-24mer mRNA constructs.

### ELISA

ELISA buffers were made as follows: washing buffer (PBS + 0.2% tween 20), blocking buffer (5% skim milk in PBS with 1% FBS and 0.2% tween 20), dilution buffer (PBS with 1% FBS and 0.2% tween 20), and stop solution (340 mL of dH_2_O with 10 mL of sulfuric acid). 96-well Corning costar assay plates were coated with 25 μl of antigen or 236 and 1512 Fab at 2 μg/ml in PBS overnight at 4 °C. After each incubation, plates were washed three times. Blocking buffer was added, incubating at room temperature for one hour. Sera were diluted 3-fold from a starting dilution of 1:500 and incubated for one hour. Antibodies were diluted 4-fold from a starting concentration of 10 μg/mL and incubated for one hour. 25 μl of peroxidase secondary antibody (Peroxidase AffiniPure Goat Anti-Mouse IgG H + L, Jackson ImmunoResearch or Peroxidase Donkey Anti-Human IgG, Jackson ImmunoResearch) was added for one hour incubation. A 1:5 dilution of TMB was added to each plate at 25 μL per well. Plates were left to stand for approximately four minutes before 25 μL per well of stop solution was added. A plate reader read at 450 nm and 570 nm, with the delta between the two readings measured and analyzed. Area under the curve (AUC) was calculated using Prism 9.1.0. Equivalence titers were determined by interpolation using standard curves of murine antibodies 14G2 and 2A10.

### Flow cytometry

Membrane-tethered constructs were produced in Freestyle 293-F cells transfected with the indicated DNAs according to manufacturer’s instructions (ThermoFisher Scientific). Two days after transfection, cells were stained with the indicated antibodies 10 nM in FACS buffer (PBS + 0.1% BSA), washed twice, labelled with Alexa Fluor 647-conjugated anti-human IgG (Jackson Immunoresearch) and analyzed on a NovoCyte 3000 (ACEA Biosciences). Data were analyzed by gating on single cells using FSC, SSC, and width in FlowJo 10.7.1 (Beckton Dickinson). The median fluorescence intensity of the Alexa-647 signal was calculated and plotted in Prism 9 for macOS (Graphpad).

### Negative stain electron microscopy

Purified samples were diluted to 0.1 mg/ml in HBS and mixed with uranyl formate for negative-stain EM using a Talos TEM. Carbon-coated copper grids (400 mesh) were glow-discharged and 10 µL of each sample was adsorbed for 2 min. Excess sample was wicked away and grids were negatively stained with 2% uranyl formate for 2 min. Excess stain was wicked away, and the grids were allowed to dry. Samples were analyzed at 80 kV with aD ThermoFisher Talos L120C transmission electron microscope, and images were acquired with a CETA 16 M CMOS camera.

### Mouse immunizations

Our standard two-dose schedule utilized female C57BL/6 J mice that were 7–8 weeks old at the time of the first immunization. Mice were immunized at week 0 and week 5 with 0.5 nanomoles of immunogen and 5 μg of SMNP adjuvant, SQ scruff of the neck. Mice were bled one week before the first immunization and one week after the second immunization for 100 μl of blood.

The only study using female C57BL/6 J mice that differed from the standard was the study utilizing RTS,S/AS01. Here, mice were immunized with 15 μg of our immunogens and 5 μg of SMNP adjuvant, SQ base of the tail. One group received RTS,S/AS01 (5 µg RTS,S adjuvanted with 5 µg of AS01_E_) via intramuscular route.

We observed no difference between administering malaria immunogens and controls SQ scruff of neck and SQ base of tail.

10 µg, 3 µg, or 1 µg of formulated mRNA/LNP was gently mixed with PBS to bring up to a total volume of 50 µl. mRNA/LNP i.m. injections were done in the left leg.

### Transgenic Sporozoite Challenge

For standard challenge, at either two weeks or five weeks after last immunization, all mice were anesthetized and challenged with 5000 P. Berghei transgenic sporozoites expressing P. falciparum CSP and luciferase through IV tail injection. Forty-two hours after challenge, mice were anesthetized and injected IP with 100 μL of D-luciferin (30 mg/mL), having been anesthetized by exposure to isoflurane. Bioluminescence in the liver was measured using an AMI HTX (Spectral Instruments). Percent inhibition of liver invasion was calculated with the following formula: Percent Inhibition=100-100*(experimental flux/adjuvant flux).

Parasitemia protocols were adapted from previously described methods^79^. Briefly, animals were anesthetized, and small bleeds were taken from mice after challenge. 1 µL of whole blood was added to PBS containing 1 μg/ml Hoechst 33258 (Thermo Fisher) and 2 µg/mL of anti-mouse CD45.2 conjugated to Alexa-647 (BioLegend) and analyzed on a ZE5 Cell Analyzer (BioRad). Live/dead stains were not used in these experiments; therefore, gated populations may contain dead cells. Data were analyzed by gating on single cells using FSC, SSC, and width in FlowJo 10.7.1 (Beckton Dickinson), followed by gating on Alexa 647 negative cells. The percentage of the GFP signal was calculated and plotted in Prism 10.1.0 for macOS (Graphpad).

At the conclusion of each experiment, mice were euthanized through CO_2_ exposure in accordance with approved guidelines. The research protocol was approved and performed in accordance with Scripps Research IACUC Protocol #22-0007.

### Statistics

Data were analyzed and plotted using Prism 10.1.0 (Graphpad). Medians and interquartile ranges were shown when appropriate. All measurements were taken using discrete samples. Statistical analysis was performed using Prism software using two-tailed Mann–Whitney assuming non-normal distribution, Kruskal-Wallis test assuming non-normal distribution, or log-rank Mantel-Cox test for survival curves. *P*-values below 0.05 (*), 0.005 (**), and 0.0005 (***) were considered significant and indicated by asterisks.

### BioRender usage

Figure 1 schematics adapted from “Membrane Proteins”, by BioRender.com (2023). Retrieved from biorender-templates.

### Ethics statement

All the experimental animal work was performed in strict compliance to the guidelines of Scripps Institutional Animal Care and Use Committee, which approved this study (under animal use protocol authorization 15-0003-4 and 22-0007-1).

## Supplementary information

Supplementary Information

## Data Availability

In addition to the provided sequences in this manuscript, the datasets used and/or analyzed in the current study are available from the corresponding author upon request.
