# Supplementary material for: mRNA delivery of circumsporozoite protein epitope-based malaria vaccines induces protection in a mouse model
Source: NPJ Vaccines. 2025 Nov 18;10:238. doi: 10.1038/s41541-025-01296-6 (PMC12627087; doi:10.1038/s41541-025-01296-6)
Supplement: Supplementary file 1 — Supplementary Information [file 41541_2025_1296_MOESM1_ESM.pdf]

Supplemental Figures

Supplemental Figures

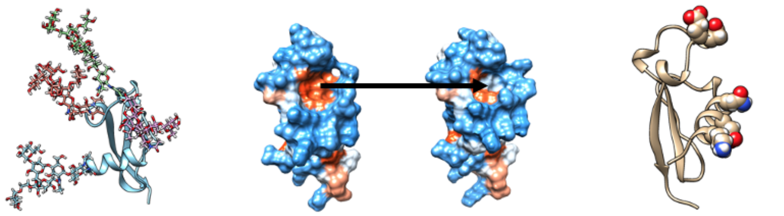

Glycan Masking   Core Filling to Eliminate Dominant Binding Mode   Alanine Resurfacing

Supplemental Figure 1: C-term MD1 Design
In order to abrogate binding to the alpha epitope, three strategies were implemented. N-linked glycosylation sites were added to the face to mask the epitope. The conserved hydrophobic pocket was modified with larger hydrophobic residues to “fill” it and prevent anti-alpha-site antibodies from utilizing it to bind. And finally, surface residues vital to the interface interactions were mutated to alanine to minimize the possible antibody interactions.

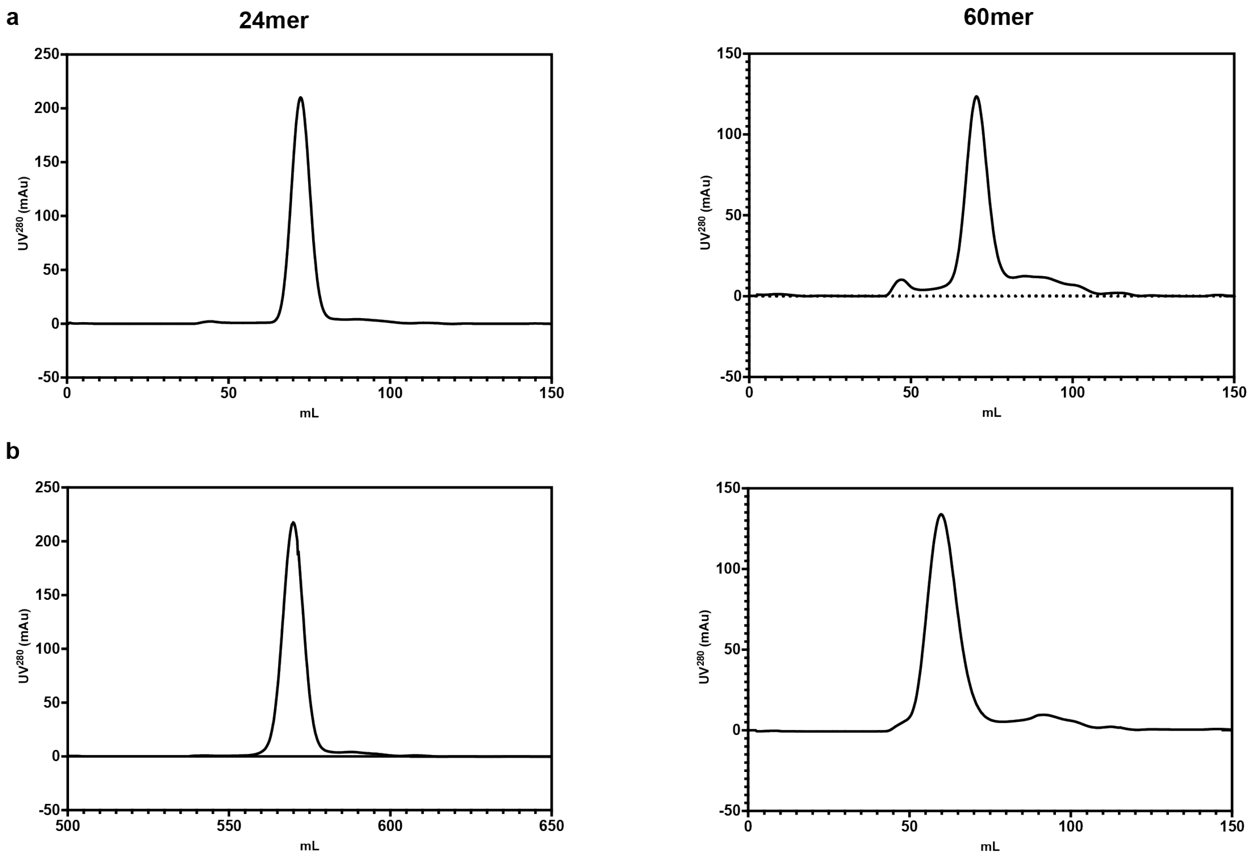

Supplemental Figure 2: SEC Profiles of C-terminal Domain Nanoparticles
SEC of C-terminal domain on 24mer and 60mer a) Wild-type b) MD1

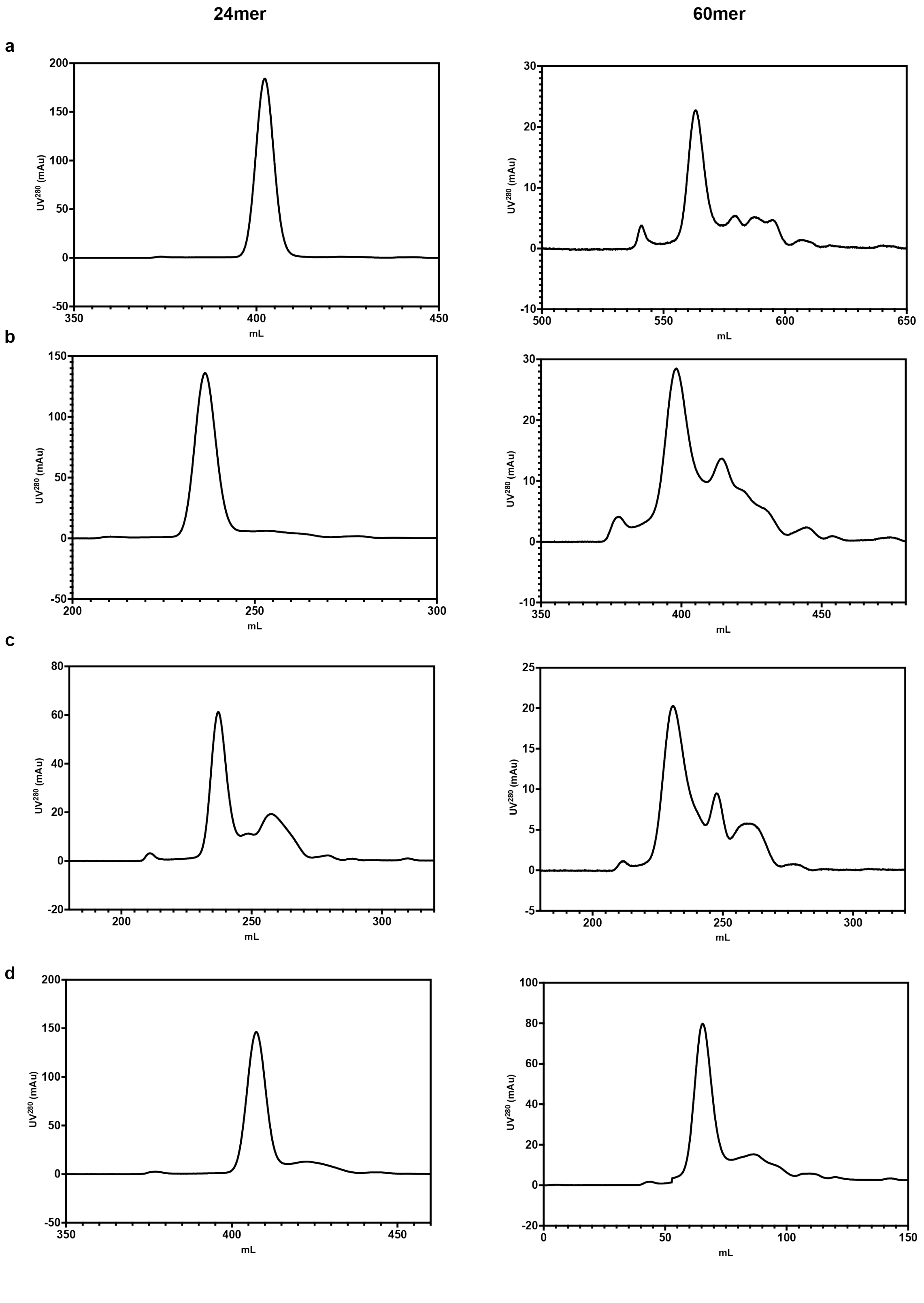

Supplemental Figure 3: SEC Profiles of Repeat Peptides Nanoparticles
SEC of repeat epitopes on 24mer and 60mer a) J2 b) J3 c) J3-R3 d) $(NANP)_6$

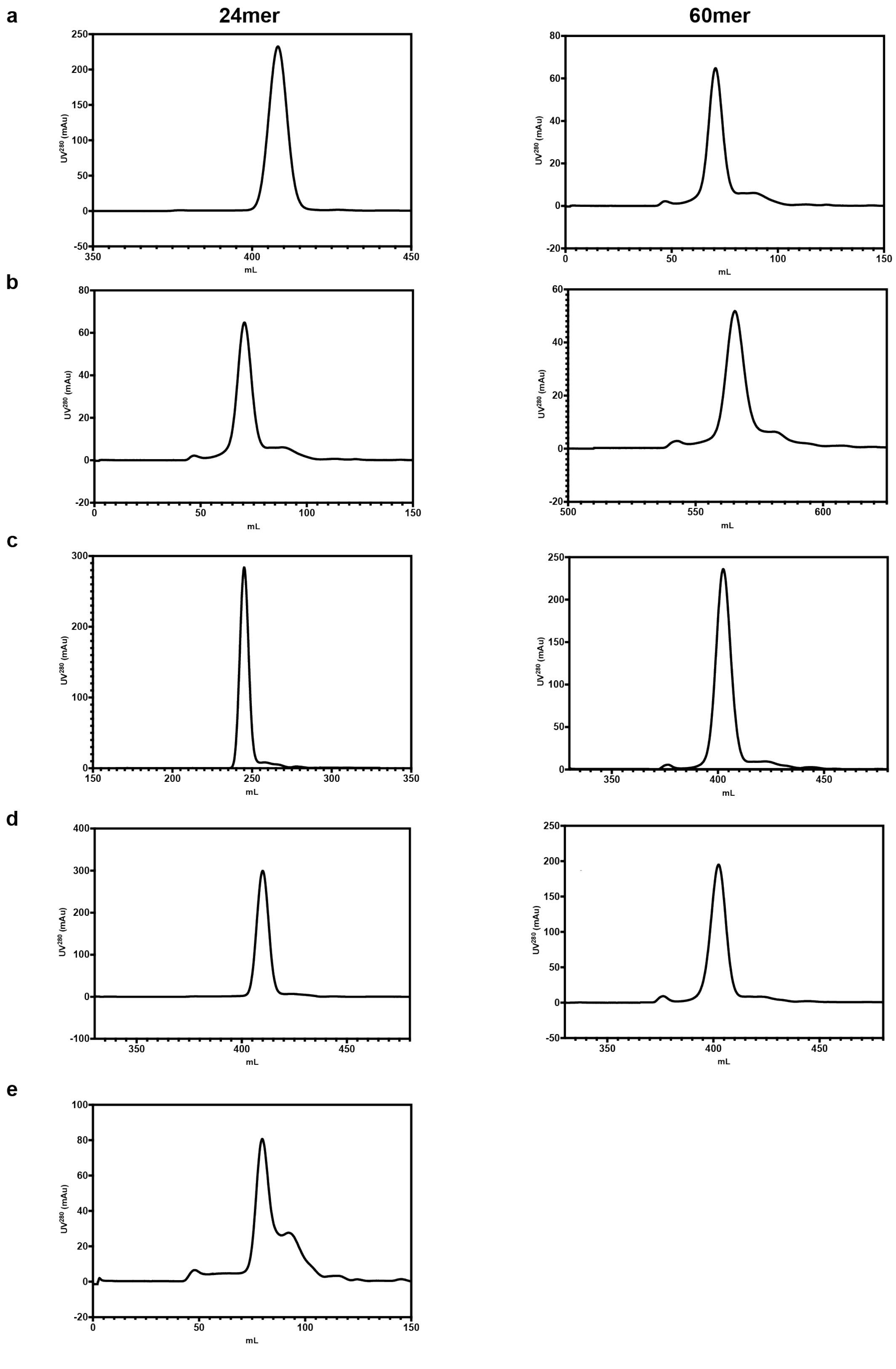

Supplemental Figure 4: SEC Profiles of N-terminal Peptides Nanoparticles
SEC of N-terminal domain epitopes on 24mer and 60mer a) P1 b) P2 c) P8 d) P9 e) P15

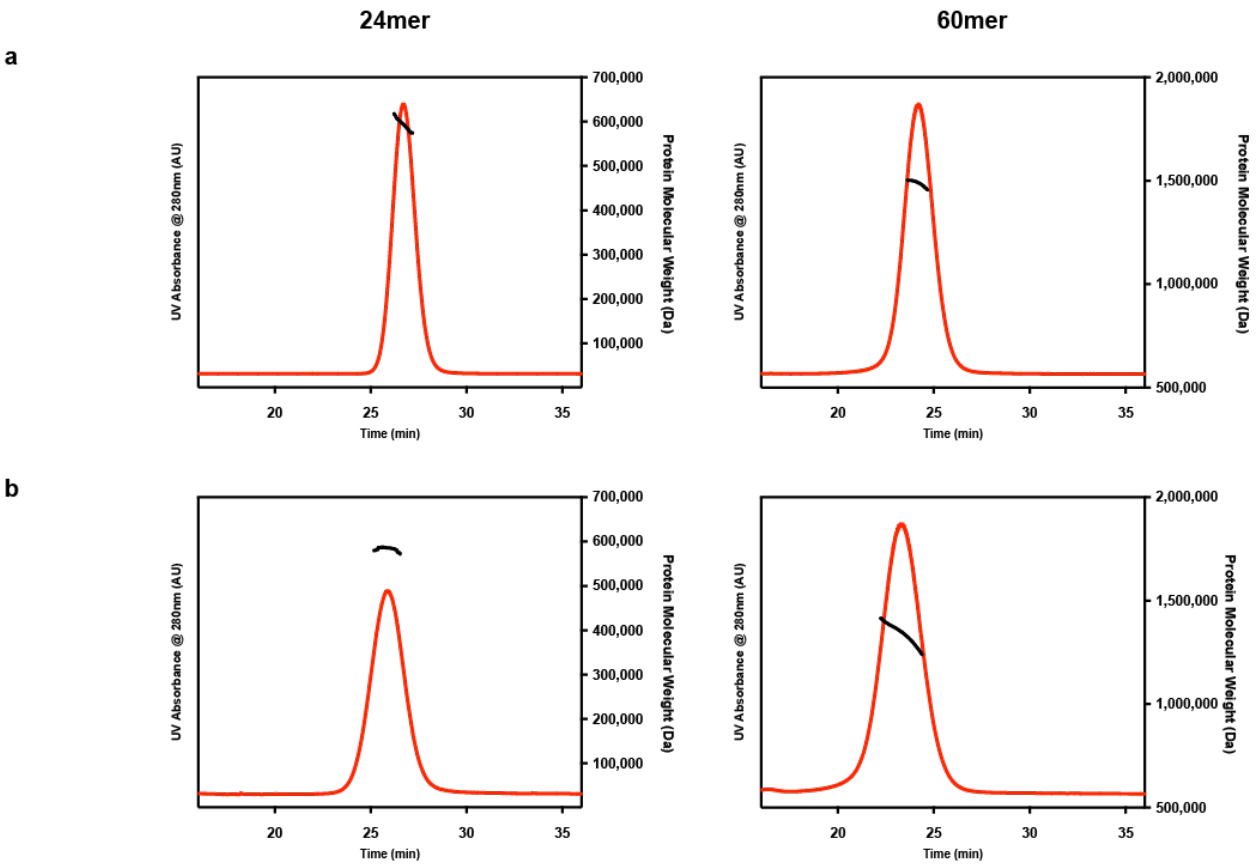

Supplemental Figure 5: SEC-MALS Profiles of C-terminal Domain Nanoparticles
SEC-MAL elution profiles showing relative UV absorbance at 280nm (red) and total protein molecular weight (black) of C-terminal domain on 24mer and 60mer a) Wild-type b) MD1

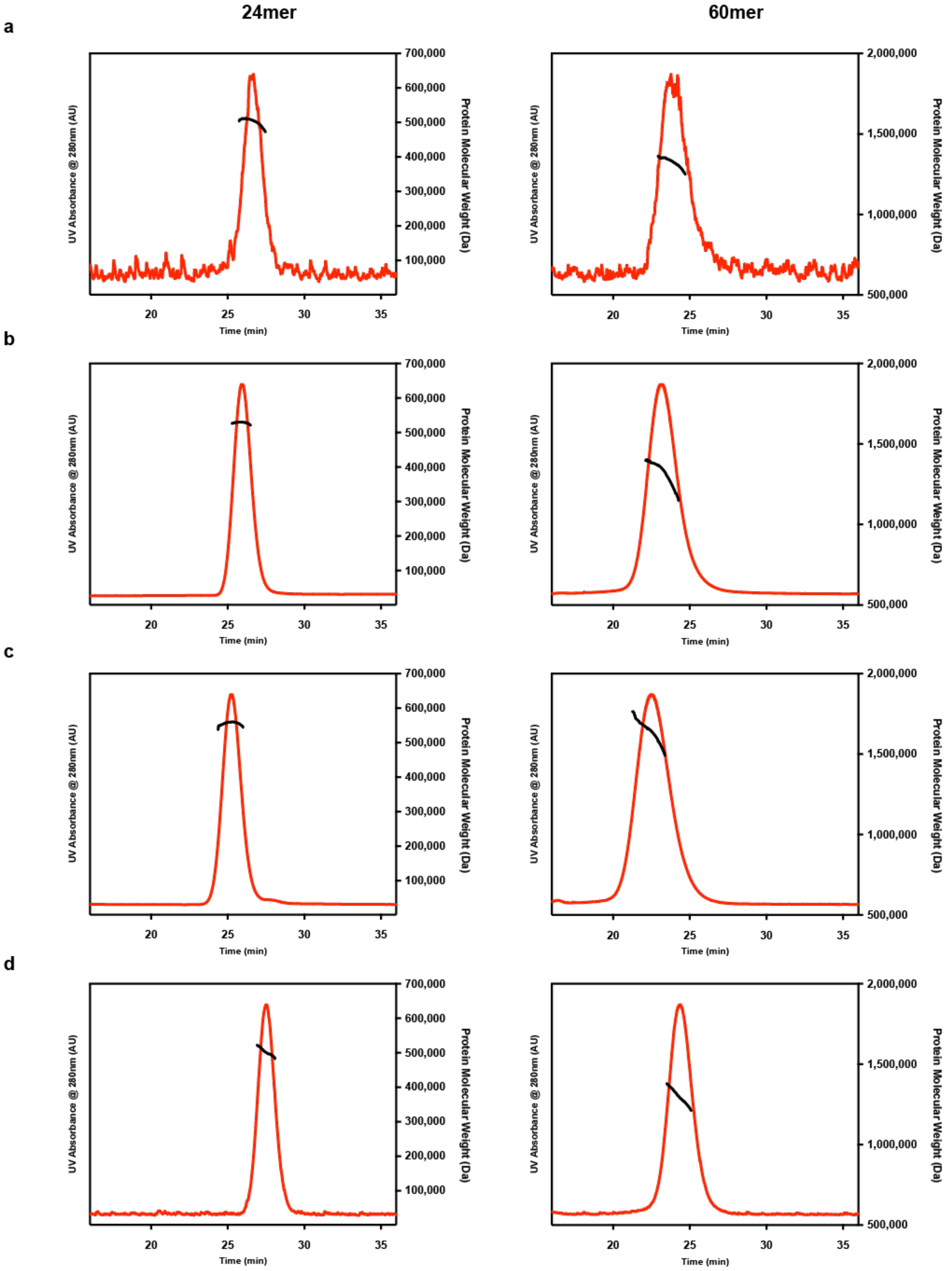

Supplemental Figure 6: SEC-MALS Profiles of Repeat Peptides Nanoparticles
SEC-MAL elution profiles showing relative UV absorbance at 280nm (red) and total protein molecular weight (black) of repeat epitopes on 24mer and 60mer a) J2 b) J3 c) J3-R3 d) $(NANP)_6$

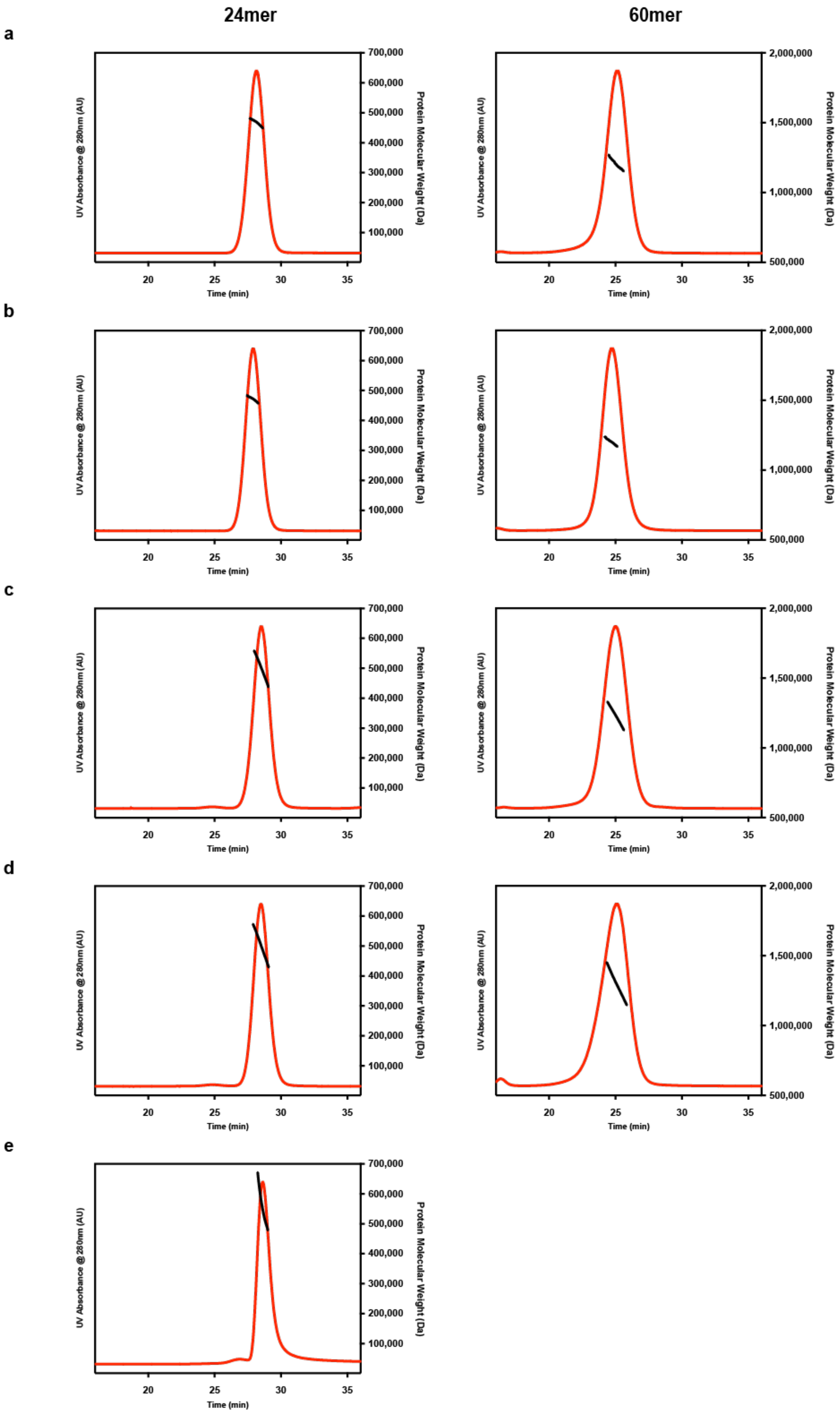

24mer
60mer
a
b
c
d
e
UV Absorbance @ 280nm (AU)
Protein Molecular Weight (Da)
Time (min)
20
25
30
35
100,000
200,000
300,000
400,000
500,000
600,000
700,000
500,000
1,000,000
1,500,000
2,000,000

Supplemental Figure 7: SEC-MALS Profiles of N-terminal Peptides Nanoparticles
SEC-MAL elution profiles showing relative UV absorbance at 280nm (red) and total protein molecular weight (black) of N-terminal domain epitopes on 24mer and 60mer a) P1 b) P2 c) P8 d) P9 e) P15

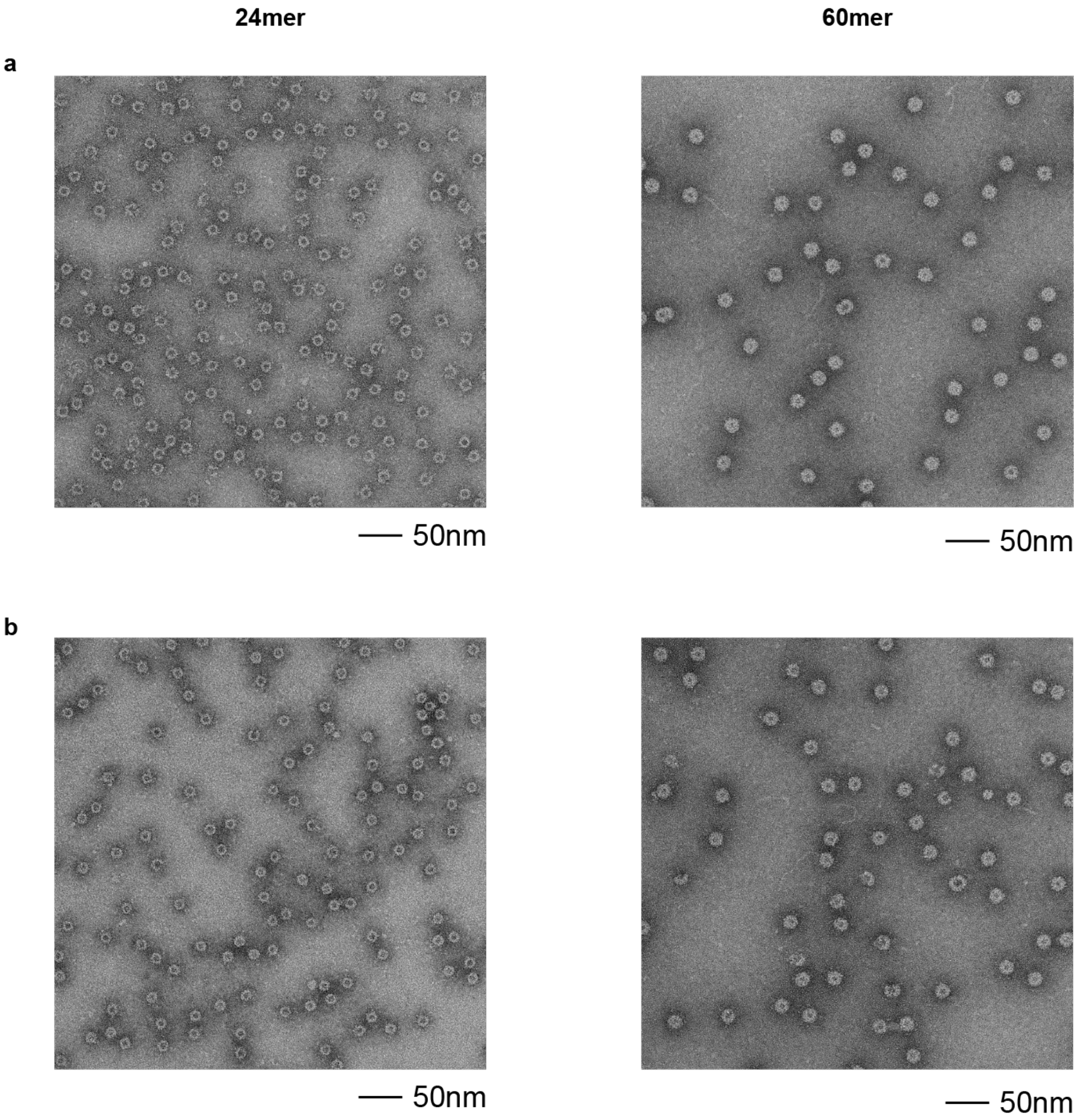

Supplemental Figure 8: Negative Stain Electron Microscopy of C-terminal Domain Nanoparticles
nsEM of C-terminal domain on 24mer and 60mer a) Wild-type b) MD1

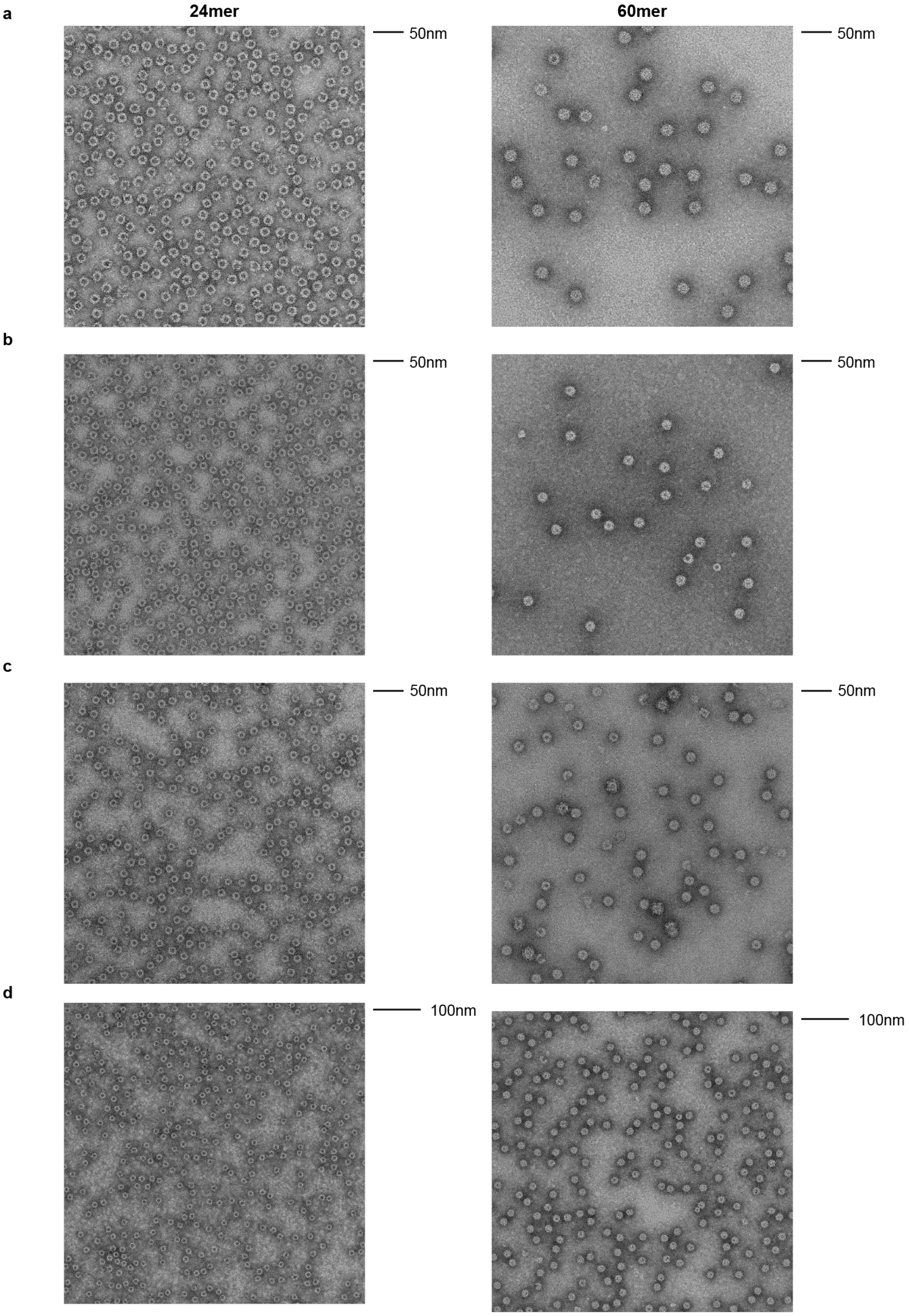
a
24mer
60mer
50nm
50nm
b
50nm
50nm
c
50nm
50nm
d
100nm
100nm

Supplemental Figure 9: Negative Stain Electron Microscopy of Repeat Peptides Nanoparticles
nsEM of repeat epitopes on 24mer and 60mer a) J2 b) J3 c) J3-R3 d) $(NANP)_6$

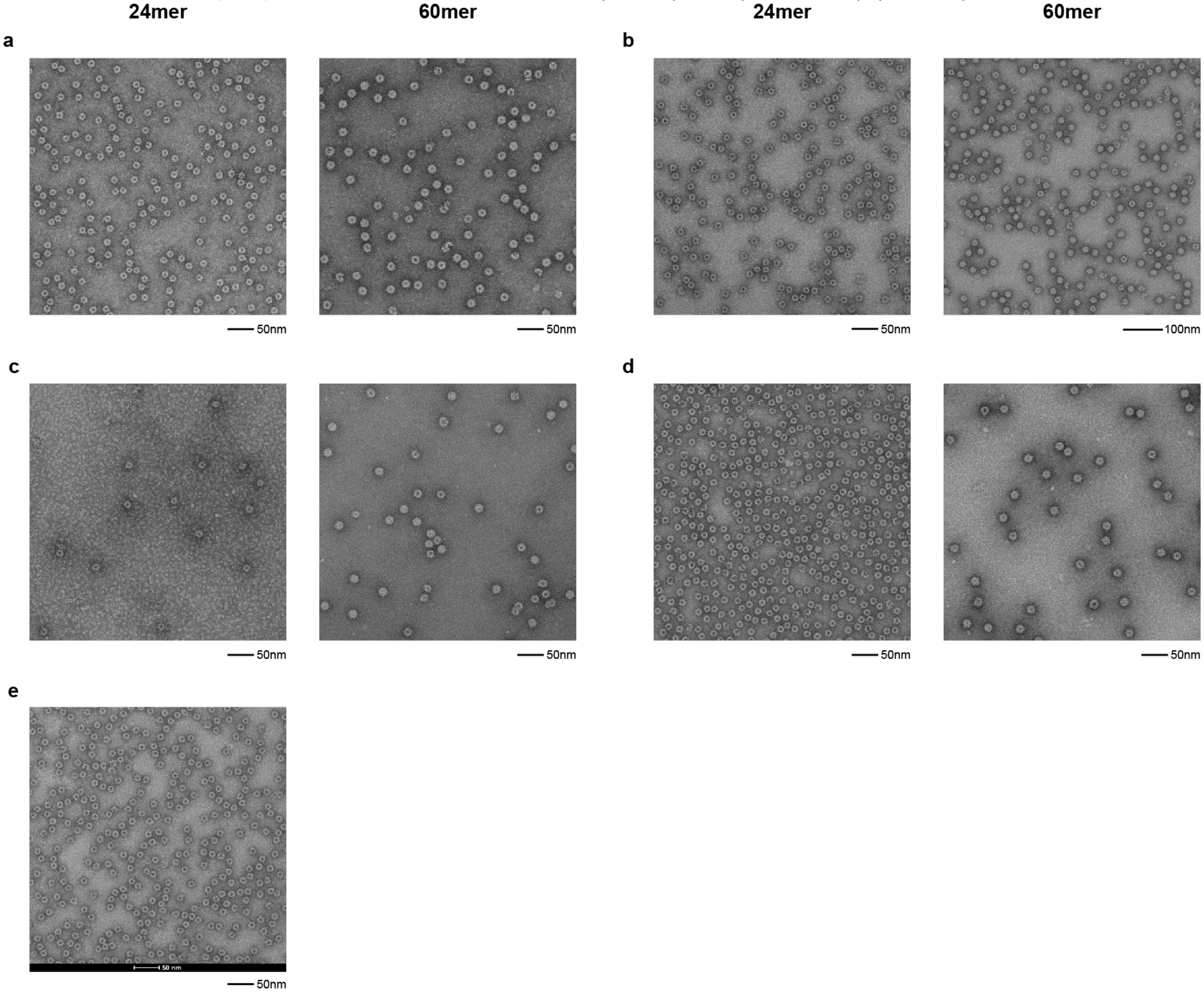

Supplemental Figure 10: Negative Stain Electron Microscopy of N-Terminal Peptides Nanoparticles
nsEM of N-terminal domain epitopes on 24mer and 60mer a) P1 b) P2 c) P8 d) P9 e) P15

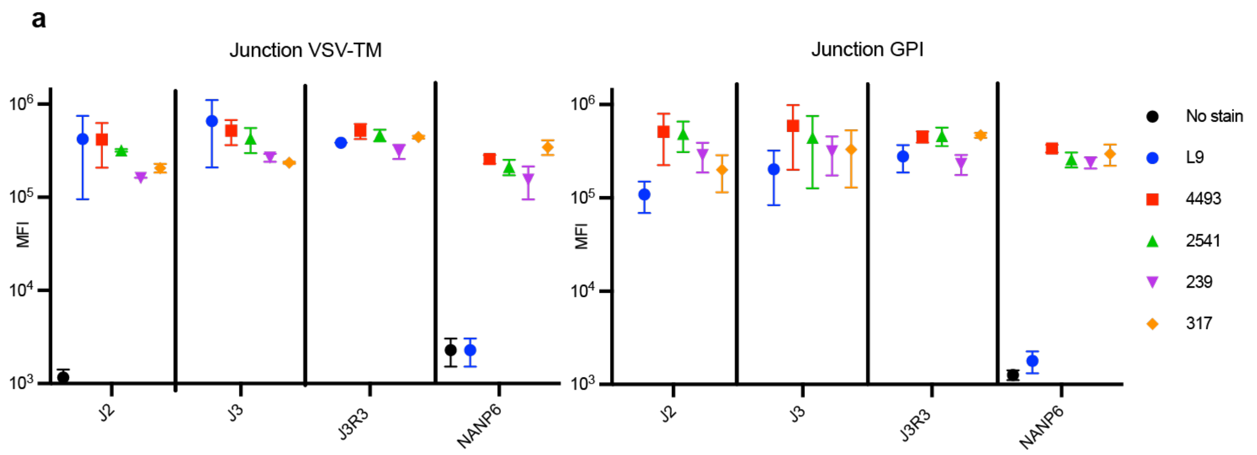

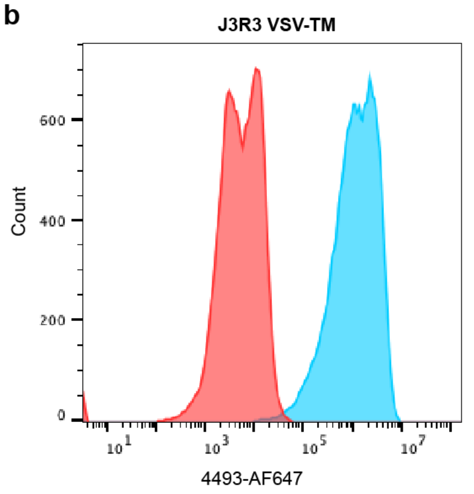

	Sample	Count	Freq. of Parent
	J3R3-VSV-TM	25800	79.0
	Naive	25772	88.0

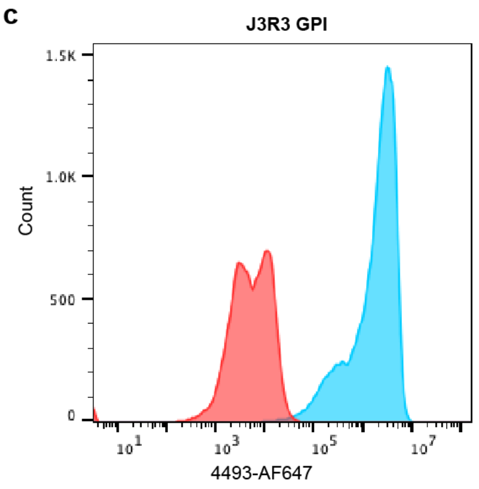

	Sample	Count	Freq. of Parent
	J3R3-GPI	36329	87.8
	Naive	25772	88.0

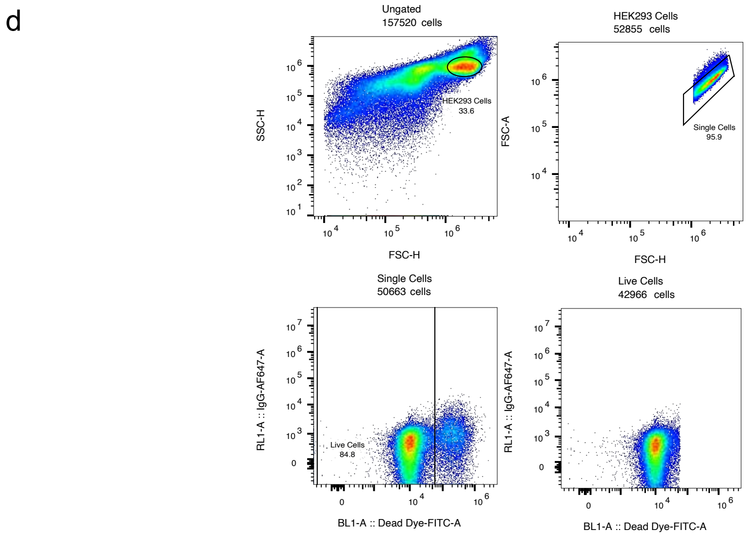

Supplemental Figure 11: Membrane-bound Platforms Characterization by FACS

a) Binding of anti-repeat antibodies 4493, 2541, 239, and 317 to repeat-displaying membrane-tethered platforms VSV-TM and GPI. Median fluorescence intensity of IgG-AF647 of Live/dead-FITC-negative values are plotted on the y-axis. Each reported measurement was from two separate experiments. Median and interquartile range are shown.

b) Histogram displaying AF647 fluorescent intensity of 4493 binding to J3R3 VSV-TM transfected cells.

c) Histogram displaying AF647 fluorescent intensity of 4493 binding to J3R3 GPI transfected cells.

d) Representative gating strategy to find HEK293 cell population from all cells, then gating for singlets, then gating for live cells. Median fluorescence intensity of IgG-AF647 was reported.

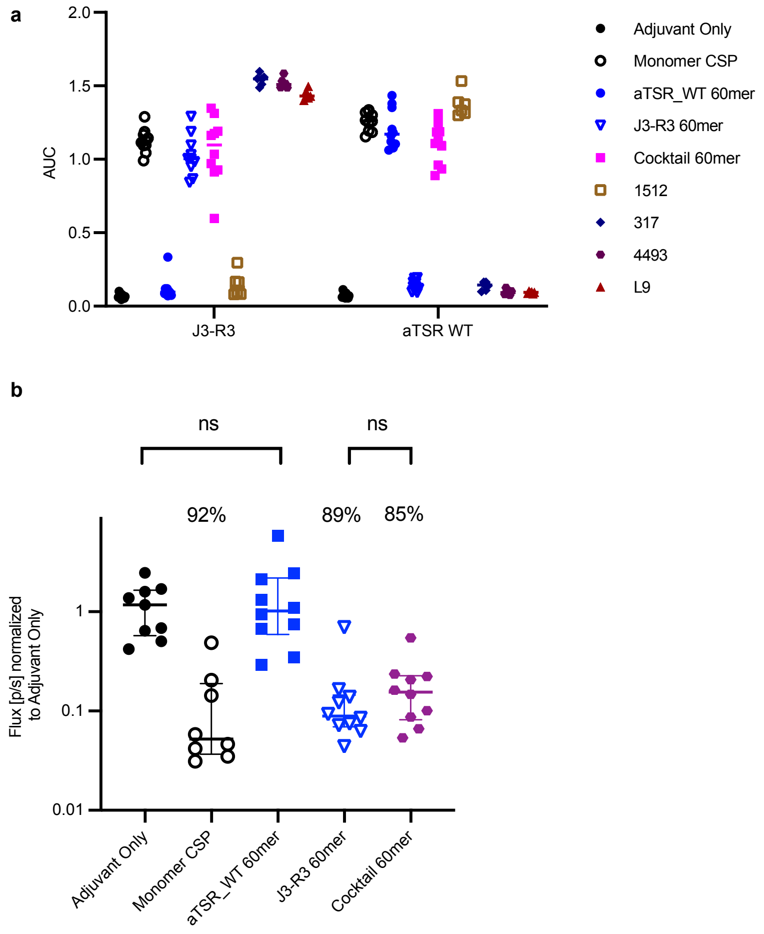

Supplemental Figure 12: Cumulative effective of Cocktail Immunization

a) Sera from immunization using J3-R3, C-terminal domain wild-type, and cocktail of the two compared in ELISA. Each symbol represents individual animals where sera was taken from a mouse one week after boost. ELISA plates were coated with J3-R3 or C-terminal domain wild-type. Median bar is shown.

b) Liver burden was assessed from immunization using J3-R3, C-terminal domain wild-type, and cocktail of the two. Each symbol represents liver fluorescence signals from individual animals. Median and interquartile range are shown. Percent protection was calculated as the reduction in signal compared to the adjuvant-only group. To compare to adjuvant-only group, two-tailed Mann-Whitney U test was performed.

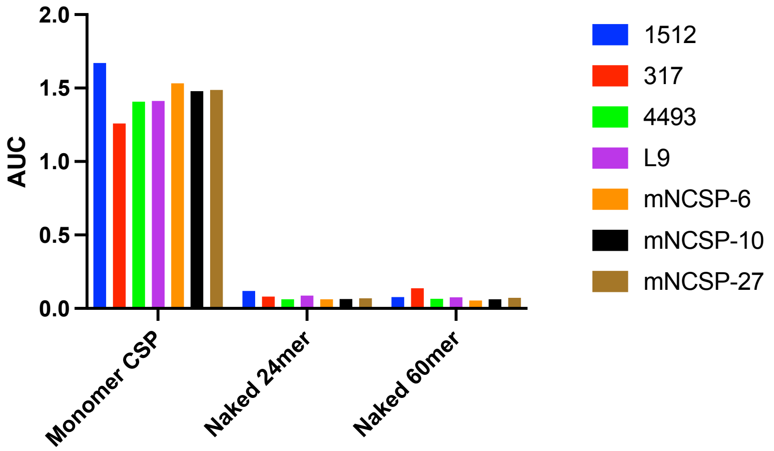

Supplemental Figure 13: Antigenicity of Naked Nanoparticles
ELISA plate coated with Monomer CSP, Naked 24mer, and Naked 60mer tested against a panel of anti-CSP malaria antibodies covering C-terminal, repeat, and N-terminal epitopes

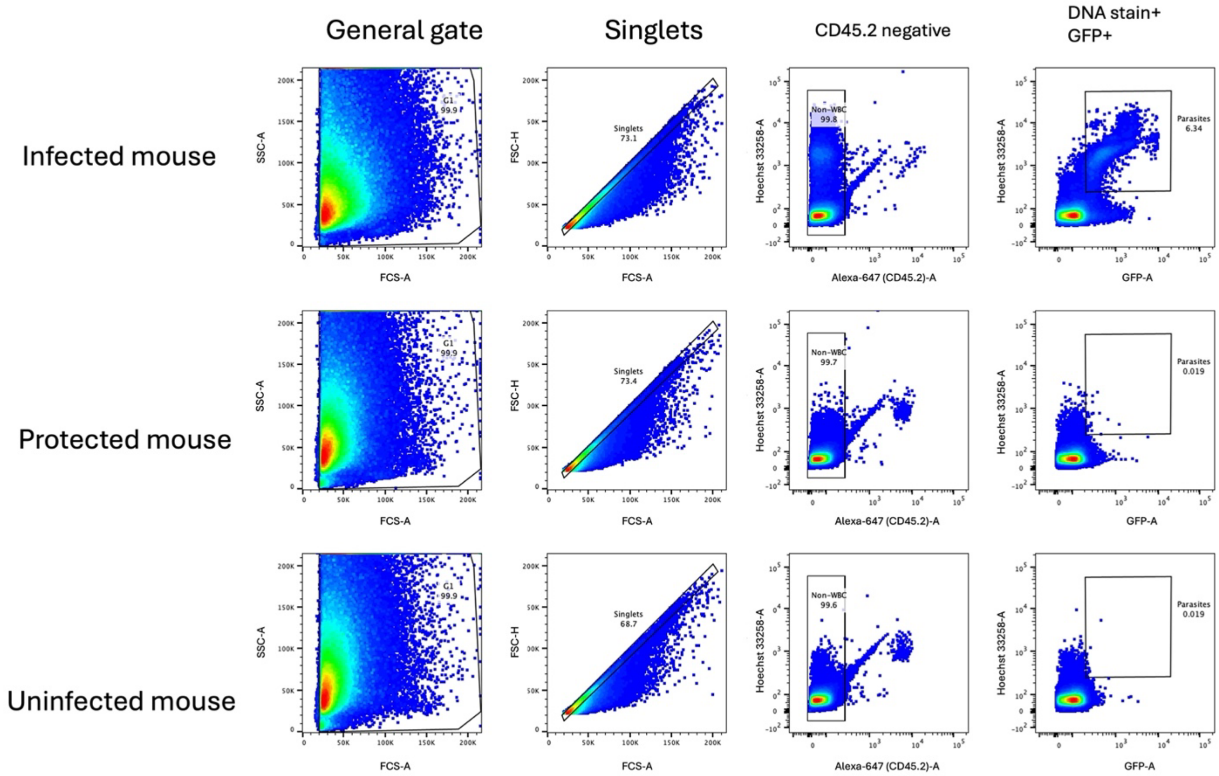

Supplemental Figure 14: Gating strategy for determination of parasitemia
Mice were infected with *P. berghei* transgenic sporozoites expressing *P. falciparum* CSP and luciferase. Whole blood was collected and labeled using an anti-CD45.2 fluorescent antibody and Hoechst 33258 DNA stain. Events were gated with a general gate followed by a singlets gate. Then, the CD45.2 negative population was selected. Parasitemia was determined by the gating for GFP and DNA positivity.

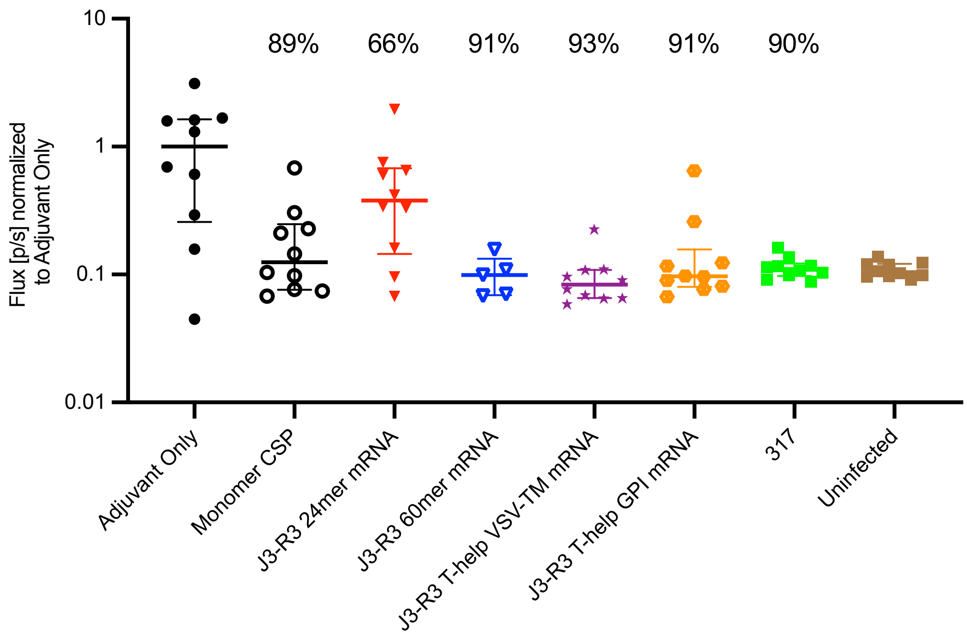

Supplemental Figure 15: Protection by vaccination with mRNA-LNPs encoding different J3R3 antigens in a low parasite challenge model.

After vaccination, mice were challenged with 300 *P. berghei* transgenic sporozoites expressing *P. falciparum* CSP and luciferase. Symbols represent individual mice (n=10). Protection was calculated as the reduction in signal compared to the adjuvant-only group and is presented above flux values as a percentage.

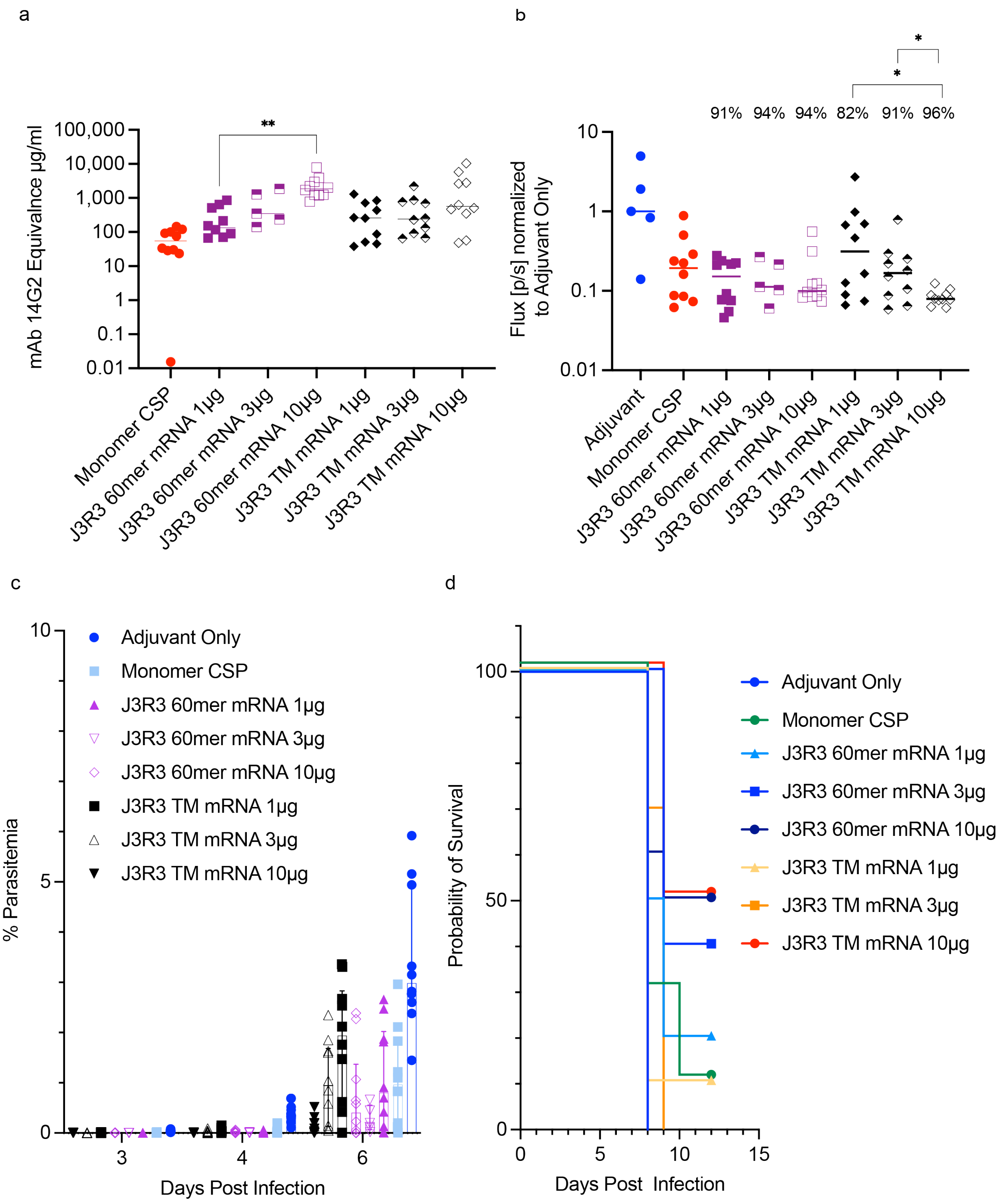

a
mAb 14G2 Equivalnce μg/ml
100,000
10,000
1,000
100
10
1
0.1
0.01
**
Monomer CSP
J3R3 60mer mRNA 1μg
J3R3 60mer mRNA 3μg
J3R3 60mer mRNA 10μg
J3R3 TM mRNA 1μg
J3R3 TM mRNA 3μg
J3R3 TM mRNA 10μg
b
Flux [p/s] normalized to Adjuvant Only
10
1
0.1
0.01
91% 94% 94% 82% 91% 96%
*
*
Adjuvant
Monomer CSP
J3R3 60mer mRNA 1μg
J3R3 60mer mRNA 3μg
J3R3 60mer mRNA 10μg
J3R3 TM mRNA 1μg
J3R3 TM mRNA 3μg
J3R3 TM mRNA 10μg
c
% Parasitemia
10
5
0
3
4
6
Days Post Infection
Adjuvant Only
Monomer CSP
J3R3 60mer mRNA 1μg
J3R3 60mer mRNA 3μg
J3R3 60mer mRNA 10μg
J3R3 TM mRNA 1μg
J3R3 TM mRNA 3μg
J3R3 TM mRNA 10μg
d
Probability of Survival
100
50
0
0
5
10
15
Days Post Infection
Adjuvant Only
Monomer CSP
J3R3 60mer mRNA 1μg
J3R3 60mer mRNA 3μg
J3R3 60mer mRNA 10μg
J3R3 TM mRNA 1μg
J3R3 TM mRNA 3μg
J3R3 TM mRNA 10μg

Supplemental Figure 16: Protection by J3R3 on nanoparticle 60mer and membrane-bound VSV-TM delivered by mRNA-LNP at different dosages in a standard sporozoite challenge model.

After vaccination, mice were challenged with *P. berghei* transgenic sporozoites expressing *P. falciparum* CSP and luciferase.

a) Circulating junctional specific IgG titers were interpolated using a 14G2 standard curve and sera from the indicated vaccination groups. Bars represent average mAb 14G2 equivalence and standard deviation respectively. Symbols represent individual mice (n=5-10) To compare to the RTS,S group, Kruskal-Wallis test was performed with $p<0.05$.

b) Liver buden is presented as flux normalized to the adjuvant only group. Bars represent median normalized flux values. Symbols represent individual mice (n=5-10). Protection was calculated as the reduction in signal compared to the adjuvant-only group and is presented above flux values as a percentage.

c) Percent composition of parasites in red blood cells in each vaccination group in a low parasite challenge model. Symbols represent individual mice (n=5-10). Bar graph indicates median and interquartile range.

d) Mouse survival following parasite challenge after vaccination with the indicated mRNA-LNPs.
